# Triclosan administration to *humanized UDP-glucuronosyltransferase 1* neonatal mice induces UGT1A1 through a dependence on PPARα and ATF4

**DOI:** 10.1016/j.jbc.2024.107340

**Published:** 2024-05-04

**Authors:** André A. Weber, Xiaojing Yang, Elvira Mennillo, Samantha Wong, Sabrina Le, Jia Ying Ashley Teo, Max Chang, Christopher W. Benner, Jeffrey Ding, Mohit Jain, Shujuan Chen, Michael Karin, Robert H. Tukey

**Affiliations:** 1 Laboratory of Environmental Toxicology, Department of Pharmacology, University of California San Diego, La Jolla, California, USA; 2 Department of Medicine, School of Medicine, University of California San Diego, La Jolla, California, USA; 3 Laboratory of Gene Regulation and Signal Transduction, Department of Pharmacology, School of Medicine, University of California San Diego, La Jolla, California, USA

**Keywords:** activating transcription factor 4 (ATF4), cytochrome P450 (CYP), constitutive androstane receptor (CAR), integrated stress response (ISR), peroxisome proliferator-activated receptor alpha (PPARα), total serum bilirubin (TSB), Uridine 5'-diphospho-glucuronosyltransferase (UGT), humanized UGT1 (*hUGT1*)

## Abstract

Triclosan (TCS) is an antimicrobial toxicant found in a myriad of consumer products and has been detected in human tissues, including breastmilk. We have evaluated the impact of lactational TCS on UDP-glucuronosyltransferase 1A1 (UGT1A1) expression and bilirubin metabolism in humanized *UGT1* (*hUGT1*) neonatal mice. In *hUGT1* mice, expression of the hepatic UGT1A1 gene is developmentally delayed resulting in elevated total serum bilirubin (TSB) levels. We found that newborn *hUGT1* mice breastfed or orally treated with TCS presented lower TSB levels along with induction of hepatic UGT1A1. Lactational and oral treatment by gavage with TCS leads to the activation of hepatic nuclear receptors constitutive androstane receptor (CAR), peroxisome proliferator-activated receptor alpha (PPARα), and stress sensor, activating transcription factor 4 (ATF4). When CAR-deficient *hUGT1* mice (*hUGT1/Car*^*−/−*^) were treated with TCS, TSB levels were reduced with a robust induction of hepatic UGT1A1, leaving us to conclude that CAR is not tied to UGT1A1 induction. Alternatively, when PPARα-deficient *hUGT1* mice (*hUGT1/Ppar*α^*−/−*^) were treated with TCS, hepatic UGT1A1 was not induced. Additionally, we had previously demonstrated that TCS is a potent inducer of ATF4, a transcriptional factor linked to the integrated stress response. When ATF4 was deleted in liver of *hUGT1* mice (*hUGT1/Atf4*^*ΔHep*^) and these mice treated with TCS, we observed superinduction of hepatic UGT1A1. Oxidative stress genes in livers of *hUGT1/Atf4*^*ΔHep*^ treated with TCS were increased, suggesting that ATF4 protects liver from excessive oxidative stress. The increase oxidative stress may be associated with superinduction of UGT1A1. The expression of ATF4 in neonatal *hUGT1* hepatic tissue may play a role in the developmental repression of UGT1A1.

Triclosan [5-chloro-2-(2,4-dichlorophenoxy)phenol; TCS] was developed as an antibacterial and antifungal agent that is now classified as an emerging environmental toxicant because of its widespread use in the commercial and industrial sectors (1, 2, 3). It currently represents one of the most abundant environmental toxicants worldwide, listed among the seven most frequent toxicants detected in surface water in the USA (1). Epidemiological studies have detected TCS in human tissue samples, including breastmilk (4, 5, 6). Chronic TCS exposure to adult mice has been associated with the development of hepatocellular carcinoma (HCC) (7), nonalcoholic fatty liver disease (NAFLD) (8, 9), and ulcerative colitis (5). However, the impact of TCS exposure on the pathophysiology of cellular function is just starting to be understood. Human exposure shows that 38% of TCS is unconjugated after oral ingestion, revealing a relatively low capacity in humans to metabolize this toxicant (10). Thus, it can be expected that individuals with a low capacity for TCS metabolism would be more susceptible to TCS-elicited toxicity.

Once TCS is absorbed, it is metabolized by the UDP-glucuronosyltransferases (UGTs) to a glucuronide [TCS glucuronide (TCS-G)], which is biologically inactive and subject to elimination (5). UGTs participate in the glucuronidation and detoxification process by converting lipophilic substrates to hydrophilic products (11). Once the glucuronides are formed, these metabolites are transported out of the cell through cellular transporters for elimination (12, 13). Among the UGTs, UGT1A1 is the only transferase capable of conjugating bilirubin and can be transcriptionally induced by several nuclear receptors (NRs) and environmental sensors (14). The ability to alter the toxic or mutagenic actions of toxicants through glucuronidation makes the UGTs an important metabolic defense system against environmental toxicants (11). Interestingly, the human *UGT1A* genes are regulated in a developmental fashion both in liver and the intestinal tract (14). Humanized *UGT1* (*hUGT1*) mice express the entire human *UGT1* locus in a *Ugt1*-null background and have served as an excellent animal model to study mechanisms involved with the induction of UGT1A1 and other human *UGT1A* genes (14, 15). In *hUGT1* mice, several NRs and transcriptional factors regulate the human *UGT1A* genes, including the pregnane-X receptor (PXR) (16), constitutive androstane receptor (CAR) (17, 18, 19), liver X receptor alpha (LXRα) (20), peroxisome proliferator-activator receptor alpha (PPARα) (21), aryl hydrocarbon receptor (AhR) (22) and the nuclear factor erythroid derived 2-related factor 2 (NRF2) (23, 24).

Previous studies have shown that TCS administration activates the integrated stress response (ISR) in the liver (8, 9). The ISR is a signaling network that helps the cell adapt to a host of variable environments which are focused on maintaining cell homeostasis (25, 26). In response to environmental and pathological conditions, including protein homeostasis defects (27), nutrient deprivation (28), viral infection (29), hypoxia (30), iron deficiency (31) and oxidative stress (27), the ISR restores balance by reprogramming gene expression. The ISR kinase sensors are RNA-dependent protein kinase–like ER kinase (PERK), double-stranded RNA-dependent protein kinase, heme-regulated eIF2a kinase, and general control non-derepressible 2. These kinases converge on the activation of activating transcription factor 4 (ATF4), a basic leucine zipper transcription factor that belongs to the activating transcription factor/cyclic AMP response element binding protein (25, 26). ATF4 has several dimerization partners that influence its regulation on patterns of gene transcription that can guide cellular fate (25, 32). It is regulated at the transcriptional, translational, and post-translational level, and moreover, its ability to interact with other transcription factors provides a further level of regulation. For example, interactions of ATF4 with C/EBP homologous protein (CHOP) promotes cell death under ER stress (32). Recent findings demonstrated that ATF4-SLC7A11 axis suppresses HCC by blocking ferroptosis (33).

The delivery of TCS to newborns through lactation can profoundly disrupt neonatal liver homeostasis, especially altering lipid metabolism (9). In the present study, we will show that lactational and oral TCS exposure to neonates dramatically affects the expression and activation of NRs and stress sensors in the liver. These studies have led us to identify unrevealed mechanisms involving ATF4 and PPARα that are linked to the induction of UGT1A1 and several *UGT1A* genes.

## Results

### Lactational TCS reduces TSB levels and induces hepatic human UGT1A1 in neonatal *hUGT1* mice

**Figure 1:**
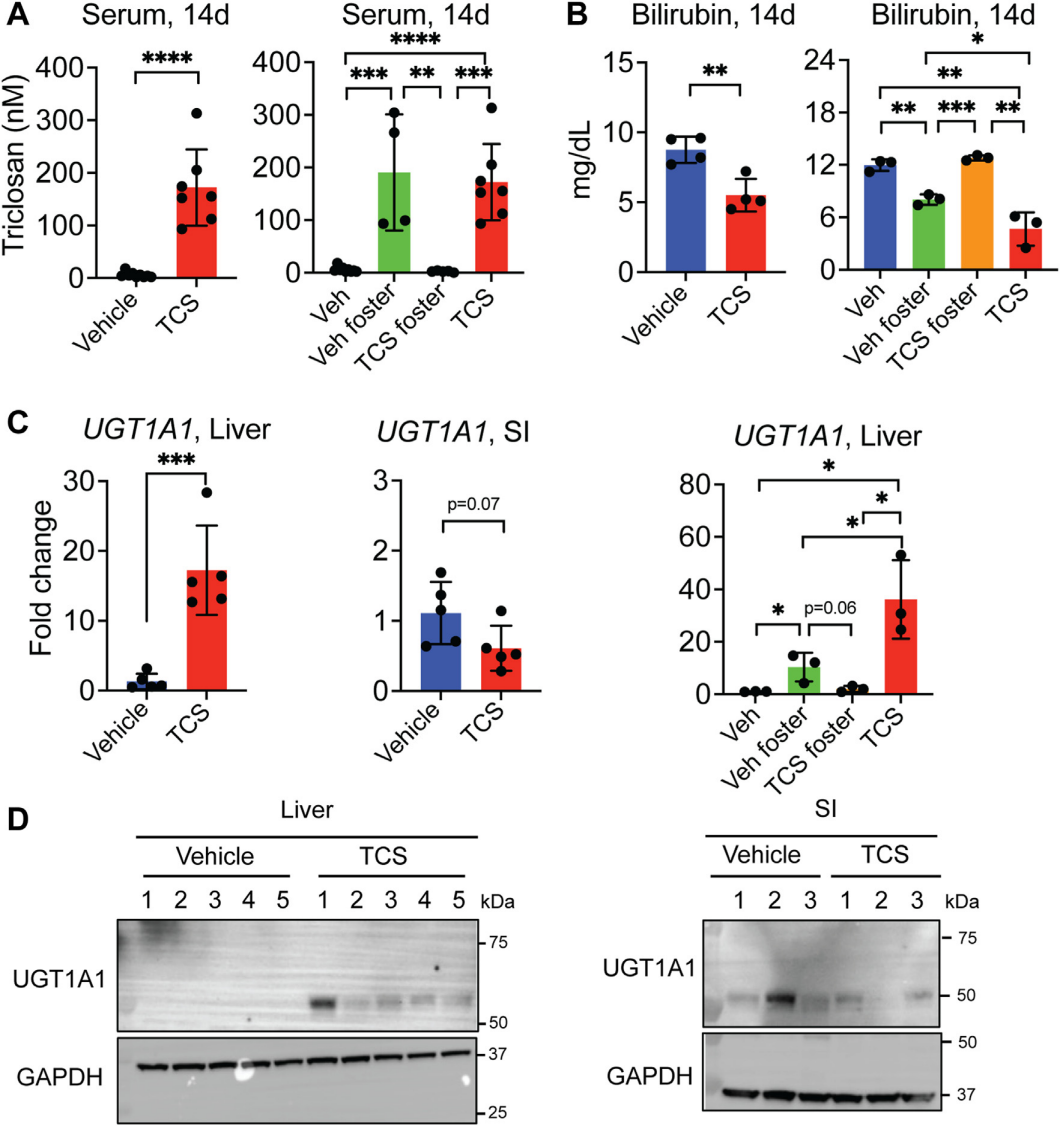
**Lactational triclosan (TCS) exposure lowers total serum bilirubin (TSB) levels with hepatic UGT1A1 induction in *hUGT1* neonates.***A*, serum levels of TCS in neonatal *hUGT1* mice that received TCS through lactation (n = 9, 7) and the levels in mice that were foster fed (n = 9, 4, 5, 7). *B*, TSB levels in neonatal mice breastfed with TCS (n = 4) and those mice that were foster fed (n = 3). *C*, Q-RT-PCR of *UGT1A1* gene expression in both liver and small intestine (SI) (n = 5) and in liver of foster fed mice (n = 3). *D*, Western Blot analysis of UGT1A1 in liver (n = 5) and SI (n = 3) of *hUGT1* mice. GAPDH served as a loading control. Results are described as mean ± S.D., determined by unpaired two-tailed Student’s *t* test; ∗*p* < 0.05, ∗∗*p* < 0.01, ∗∗∗*p* < 0.001 and ∗∗∗∗*p* < 0.0001. Individual *p* value was listed in Table S2. *hUGT1*, humanized *UGT1*; UGT1A1, UDP-glucuronosyltransferase 1A1.

Pregnant *hUGT1* mice were fed vehicle chow containing vehicle dimethyl sulfoxide (DMSO) or TCS with the newborn mice being breastfed for 14 days. After 14 days, neonatal mice were sacrificed to collect blood, liver, and small intestine. TCS in serum of breast-fed neonates was measured by LC-MS/MS analysis. The concentration of TCS in serum at 14 days of exposure was 172 ± 72 nM (Fig. 1*A*). These concentrations are like those reported in blood in human samples (10, 34). Additionally, we performed foster studies to examine if TCS could be transferred through lactation to vehicle fed neonates. We first transferred newborns who were born to a dam fed by vehicle chow and placed with a dam fed with TCS chow (Veh foster) 7 days after birth. We also transferred neonates born to a dam fed with TCS chow to a dam fed with vehicle chow (TCS foster). These mice were allowed to breast feed until they were 14 days old. When we examined Veh foster neonates, they showed an increase in serum TCS after 7 days. Interestingly, when we examined TCS foster neonates, these mice showed a total depletion in serum TCS (Fig. 1*A*). Detection of TCS-G, the main TCS metabolite, followed the same trend in TCS exposed groups (Fig. S1*A*). Newborn *hUGT1* mice receiving TCS through lactation presented TSB levels that were much lower than *hUGT1* neonates from vehicle breastfed mice (Fig. 1*B*). Additionally, the fostering study showed a decrease in TSB levels in Veh foster (Fig. 1*B*), indicating that lactational delivery of TCS leads to induction of UGT1A1. Gene expression and protein analysis in liver showed induction of UGT1A1 only in hepatic tissue (Fig. 1, *C* and *D*). The fostering experiment showed an increase in UGT1A1 in Veh foster, confirming that TCS is transferred through lactation (Fig. 1*C*).

### Lactational TCS transfer to newborns alters NR target gene expression in liver

**Figure 2:**
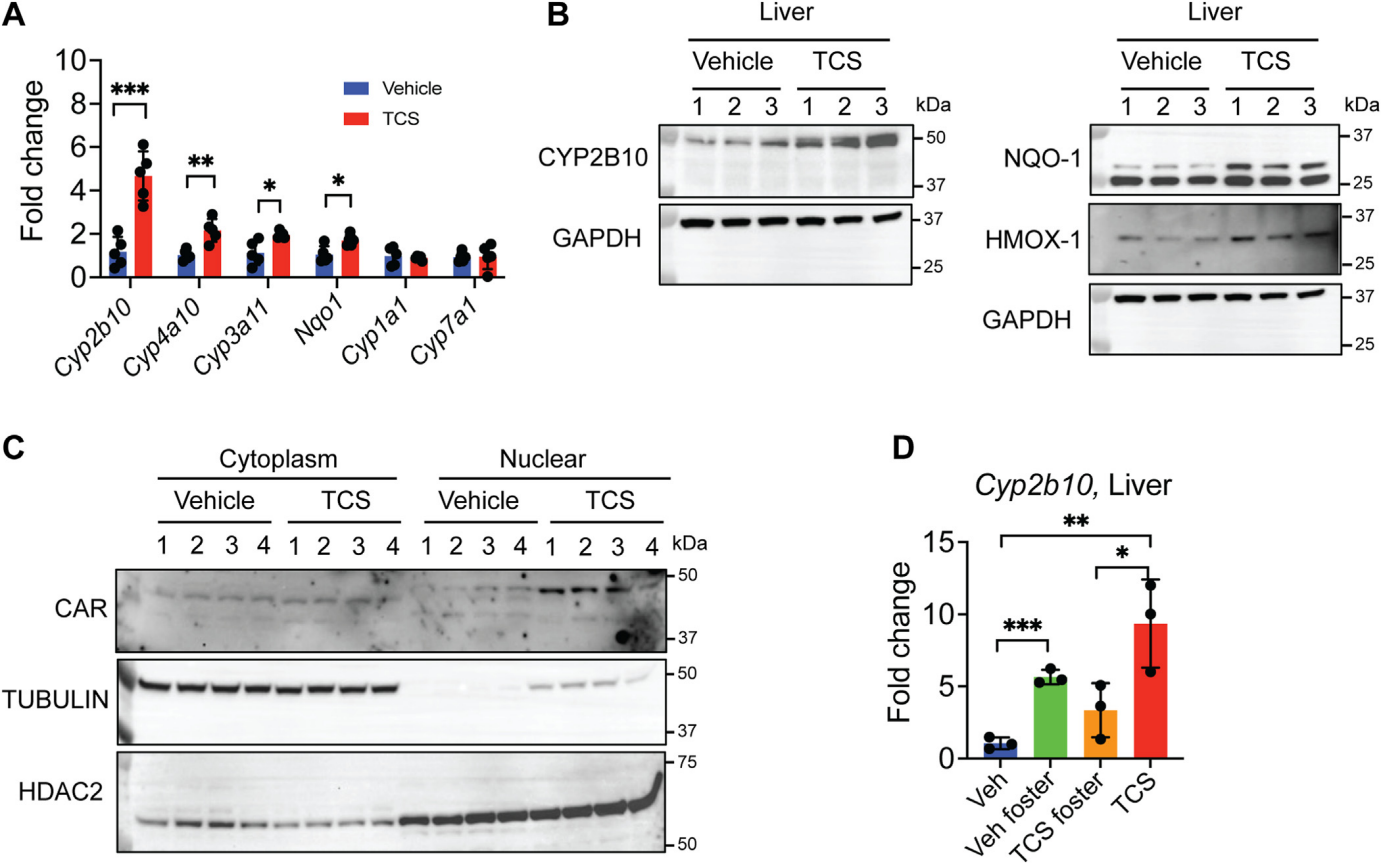
**TCS exposure through lactation induces genes targeted by nuclear receptors.***A*, RT-qPCR of several nuclear receptors and environmental sensor target genes in *hUGT1* neonatal mice receiving TCS exposure through lactation (n = 5). *B*, Western Blot analysis of CYP2B10, NQO-1, and HMOX1 in liver. GAPDH served as a loading control (n = 3). *C*, liver cell fractionation analysis by Western Blot of CAR. Tubulin and histone deacetylase 2 were used as loading controls for cytoplasm and nucleus, respectively (n = 4). *D*, RT-qPCR of *Cyp2b10* in foster-fed mice (n = 3). Results are described as mean ± S.D., determined by unpaired two-tailed Student’s *t* test; ∗*p* < 0.05, ∗∗*p* < 0.01, and ∗∗∗*p* < 0.001. Individual *p* value was listed in Table S3. CAR, constitutive androstane receptor; hUGT1, humanized *UGT1*; NQO-1, NAD(P)H quinone dehydrogenase species; RT-qPCR, reverse transcription-quantitative PCR; TCS, triclosan; UGT1, UDP-glucuronosyltransferase 1.

Previous findings have demonstrated that lactational exposure to neonates as well as long-term TCS exposure to adults can significantly impact liver pathophysiology (7, 8, 9). Following exposure to TCS through lactation for 14 days, the expression of the *Cyp2b10* gene, a target gene of activated CAR, was induced 4-fold when compared to control-treated neonates (Fig. 2*A*). Furthermore, CYP2B10 protein levels were also increased in livers of TCS-treated mice (Fig. 2*B*). Cell fractionation of liver tissue revealed an accumulation of CAR protein levels in nuclear extracts of neonatal mice breastfed with TCS (Fig. 2*C*), while the foster study showed an increase in *Cyp2b10* gene expression in Veh foster neonatal mice (Fig. 2*D*). In addition, NRF2 target genes were also induced in liver following TCS treatment. NRF2 is the master sensor of oxidative stress (35). Reverse transcription-quantitative PCR (RT-qPCR) analysis showed an increase in *Nqo1*, a target NRF2 gene (Fig. 2*A*). In addition, Western Blot analysis showed higher protein levels of NQO1 and HMOX1 in the liver of TCS-treated mice (Fig. 2*B*). In previous studies, we had demonstrated that the generation of reactive oxygen species (ROS) by isothiocyanates can also activate CAR. The activation of CAR and NRF2 suggests that lactational TCS exposure is leading to the production of ROS and oxidative stress in the liver of neonates (19, 35). It is known that overexpression of hepatic NRF2 can lead to enlargement of the liver, a process called hepatomegaly (35). Indeed, exposure to TCS leads to an increase in liver weight (7). Furthermore, RT-qPCR analysis revealed that *Cyp4a10* and *Cyp3a11*, which are regulated by PPARα and PXR, respectively, are also upregulated by lactational TCS (Fig. 2*A*). These results corroborate previous studies demonstrating that TCS can regulate PPARα and PXR (8, 9, 36). Further analysis demonstrated that lactational TCS has no effect on *Cyp7a1*, an LXRα target gene, and *Cyp1a1*, an AhR target gene (Fig. 2*A*). Thus, exposure by TCS through lactation has a significative impact on NR activation in the neonatal liver.

### Role of NRs CAR and PPARα in the regulation of UGT1A1

**Figure 3:**
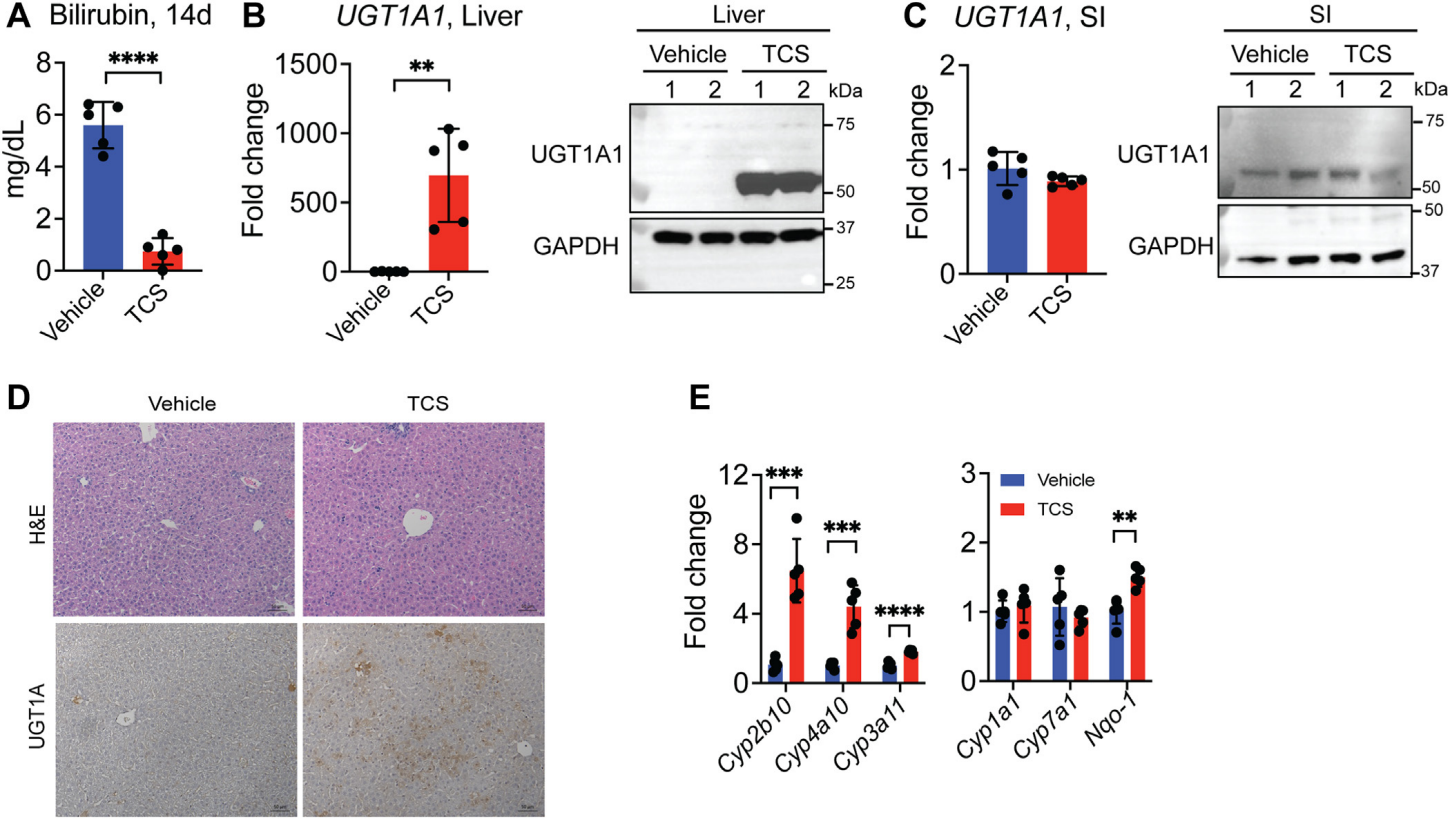
**Oral treatment with TCS strongly induces hepatic UGT1A1 in *hUGT1* neonates.** Ten-day-old *hUGT1* mice were administered TCS (50 mg/kg) by oral administration each day for 4 consecutive days. *A*, TSB levels in *hUGT1* neonatal mice treated with TCS by oral gavage (n = 5). *B*, RT-qPCR analysis of *UGT1A1* gene expression (n = 5) and Western Blot of UGT1A1 (n = 2) in liver from *hUGT1* neonatal mice treated with TCS by oral gavage. *C*, RT-qPCR analysis of *UGT1A1* gene expression (n = 5) and Western Blot of UGT1A1 (n = 2) in small intestine (SI) of *hUGT1* neonatal mice treated with TCS by oral gavage. *D*, H&E staining and IHC for UGT1A protein of 14-day-old mice treated with TCS by oral gavage (n = 3) Scale bars= 50 μm. *E*, RT-qPCR of NR and environmental sensor target genes in *hUGT1* neonatal mice treated with TCS by oral gavage (n = 5). Results are described as mean ± S.D., determined by unpaired two-tailed Student’s *t* test; ∗∗*p* < 0.01, ∗∗∗*p* < 0.001, and ∗∗∗∗*p* < 0.0001. Individual *p* value was listed in Table S4. hUGT1, humanized *UGT1*; NR, nuclear receptor; RT-qPCR, reverse transcription-quantitative PCR; TCS, triclosan; UGT1A1, UDP-glucuronosyltransferase 1A1.

We have previously reported that human UGT1A1 and several other *UGT1A* genes are induced following activation of NRs as well as several environmental sensors (14, 37). When we treated 10-day-old *hUGT1* neonates with a range of TCS concentrations from 25 to 100 mg/kg, a dose-dependent reduction in TSB levels and a dose-dependent increase in *UGT1A1*, *Cyp2b10*, and *Cyp4a10* gene expression was observed (Fig. S2, *A–E*). Our previous studies have linked serum TSB levels in *hUGT1* mice directly with the expression of liver and/or intestinal UGT1A1 (14, 15). We treated neonatal *hUGT1* mice with TCS (50 mg/kg) by oral gavage for 4 consecutive days from 10 days old until 13 days old. When *hUGT1* mice are 13 days old, they display elevated levels of serum TSB. The gavage treatment had similar results to our breastfeeding studies; however, the acceleration of TSB clearance was more dramatic. The TSB levels dropped (Fig. 3*A*) along with a dramatic induction of *UGT1A1*, *UGT1A3*, and *UGT1A4* gene expression in liver (Figs. 3, *B* and *C* and S3). Histological analysis of liver from control and TCS-treated *hUGT1* neonates had a normal appearance with no morphological alterations. By contrast, IHC staining with an anti-UGT1A antibody revealed a significant increase in UGT1A staining in livers of neonatal mice treated with TCS (Fig. 3*D*).

In addition, oral gavage of TCS resulted in a robust increase in *Cyp2b10* and *Cyp4a10* gene expression in liver and a slight increase in *Cyp3a11* and *Nqo-1* gene expression (Fig. 3*E*). This result indicates strong activation of CAR and PPARα with modest activation of PXR and NRF2, respectively. Target genes of LXRα (*Cyp7a1*) and the AhR (*Cyp1a1*) were unaltered by oral TCS treatment (Fig. 3*E*). Since CAR and PPARα target genes were induced by TCS, we evaluated the role of CAR and PPARα in the induction of human UGT1A1 by TCS. DAVID-based GO analysis of RNA-seq data showed that the top five significant changes in biological processes upon TCS treatment were monocarboxylic acid processes, long-chain fatty acid processes, peroxisomal protein import, chemical carcinogenesis, and mitochondrial fatty acid beta-oxidation (Fig. S4*A*). Furthermore, we performed a volcano plot between vehicle and TCS treatment. The top nine genes upregulated by oral TCS were *Cyp* genes (*Cyp2b10* and *Cyp2c50*), carboxylesterases (*Ces1f*, *Ces1g*, *Ces2a*, and *Ces1d*), PPARα target genes (*Ehhadh* and *Acaa1b*), and an Nrf2 target gene (*Abcc3*) (Fig. S4*B*).

**Figure 4:**
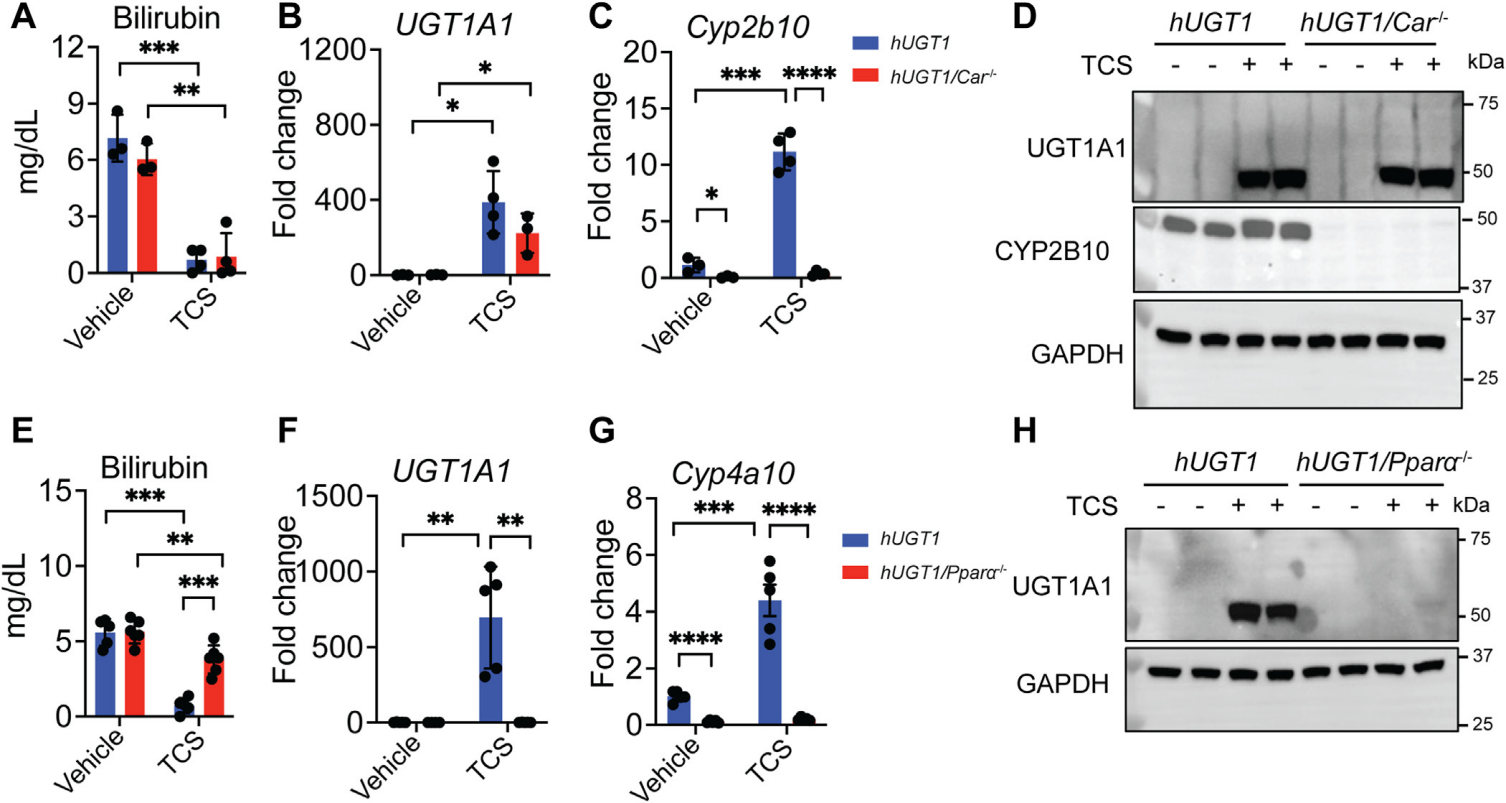
**The dependency of PPARα toward TCS-mediated induction of UGT1A1.***hUGT1*, *hUGT1/Car*^−/−^, and *hUGT1/Pparα*^−/−^ neonates at 10-days-old were orally treated for 4 consecutive days with vehicle or 50 mg/kg TCS. After 4 days, livers and blood were collected. *A*, TSB levels in *hUGT1* and *hUGT1/Car*^*−/−*^ mice. *B*, RT-qPCR of the *UGT1A1* gene in *hUGT1* and *hUGT1/Car*^*−/−*^ mice. *C*, RT-qPCR of the *Cyp2b10* gene were performed in *hUGT1* and *hUGT1/Car*^−/−^ neonates (n = 3, 3, 4, 3). *D*, Western Blot analysis of hepatic UGT1A1 and CYP2B10 were performed in *hUGT1* and *hUGT1/Car*^−/−^ neonates. GAPDH served as a loading control (n = 2). *E*, TSB levels in *hUGT1* and *hUGT1/Ppar*^*−/−*^ mice. *F*, RT-qPCR of *UGT1A1* gene expression in *hUGT1* and *hUGT1/Ppar*^*−/−*^ mice. *G*, RT-qPCR of *Cyp4a10* was performed in *hUGT1* and *hUGT1/Pparα*^−/−^ neonates (n = 5, 4, 5, 5). *H*, Western Blot analysis of hepatic UGT1A1 from *hUGT1* and *hUGT1/Pparα*^−/−^ neonates. GAPDH served as a loading control (n = 2). Results are described as mean ± S.D., determined by unpaired two-tailed Student’s *t* test; ∗*p* < 0.05, ∗∗*p* < 0.01, ∗∗∗*p* < 0.001, and ∗∗∗∗*p* < 0.0001. Individual *p* value was listed in Table S5. hUGT1, humanized *UGT1*; PPARα, peroxisome proliferator-activated receptor alpha; RT-qPCR, reverse transcription-quantitative PCR; TCS, triclosan; TSB, total serum bilirubin; UGT1A1, UDP-glucuronosyltransferase 1A1.

To examine the possibility that activated CAR is inducing hepatic UGT1A1 after TCS treatment, 10-day-old *hUGT1/Car*^*−/−*^ neonatal mice were treated by oral gavage followed by analysis of gene expression, protein, and TSB levels. The TSB levels were reduced in *hUGT1/Car*^*−/−*^ mice (Fig. 4*A*) treated with TCS with a robust induction of the *UGT1A1* gene (Fig. 4*B*) and protein expression levels (Fig. 4*D*). The low levels of *Cyp2b10* transcripts in *hUGT1/Car*^*−/−*^ confirmed that this gene is dependent on CAR (Fig. 4*C*). The reduction in TSB levels and induction of hepatic UGT1A1 in *hUGT1/Car*^*−/−*^ mice after TCS treatment indicates that CAR is not a key player involved in TCS-induced neonatal UGT1A1 expression.

A similar approach was developed to examine if PPARα was linked to the induction of UGT1A1 since TCS treatment resulted in a robust increase in *Cyp4a10* gene transcripts in liver of *hUGT1* neonatal mice. When TCS was administered to *hUGT1/Pparα*^*−/−*^ mice, there was no induction of hepatic UGT1A1 or metabolism of serum bilirubin. After oral gavage of TCS to *hUGT1*/*Pparα*^*−/−*^ mice, TSB levels presented only a slight drop in *hUGT1/Pparα*^*−/−*^-treated mice compared with those that received only vehicle (Fig. 4*E*). Furthermore, in livers of *hUGT1/Pparα*^*−/−*^ mice, no induction of hepatic UGT1A1 was detected (Fig. 4, *F* and *H*). The low levels of *Cyp4a10* transcripts in *hUGT1/Pparα*^*−/−*^ mice confirms that this gene is dependent on PPARα activation (Fig. 4*G*). This finding confirms that PPARα is a key NR that becomes activated following TCS treatment and drives the expression of the *UGT1A1* gene.

### The role of hepatic ATF4 in the induction hepatic UGT1A1 following TCS treatment

**Figure 5:**
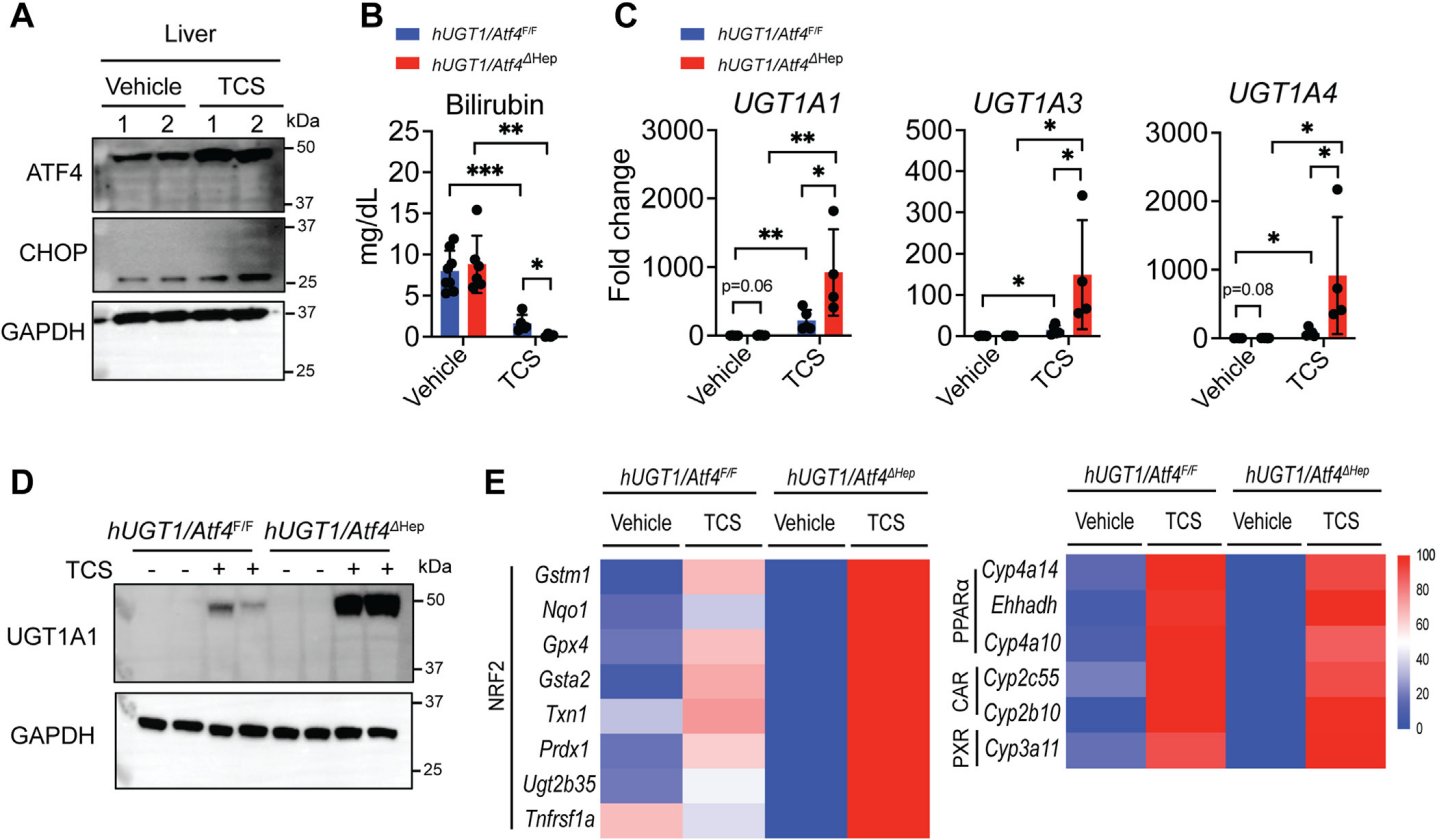
**ATF4 deletion contributes toward TCS-mediated induction of UGT1A1 and other human UGT1A genes.***hUGT1/Atf4*^*F/F*^ and *hUGT1/Atf4*^*ΔHep*^ neonatal mice at 10 days old were orally treated by 4 consecutive days with vehicle or 50 mg/kg TCS. After 4 days, livers and blood were collected. *A*, Western Blot analysis of ATF4 and CHOP in liver of *hUGT1* treated with 4 consecutive days with vehicle or 50 mg/kg of TCS (n = 2). GAPDH served as a loading control. *B*, TSB levels of *hUGT1/Atf4*^*F/F*^ and *hUGT1/Atf4*^*Δhep*^ neonatal mice treated with TCS by oral gavage (n = 8, 6, 5, 4). *C*, RT-qPCR of hepatic *UGT1A1*, *UGT1A3*, and *UGT1A4* gene expression (n = 5, 6, 5, 4). *D*, Western Blot analysis of hepatic UGT1A1 (n = 2). GAPDH served as a loading control. *E*, heatmaps of the average expression of NRF2, PPARα, CAR, and PXR target genes. Results are described as mean ± S.D., determined by unpaired two-tailed Student’s *t* test; ∗*p* < 0.05, ∗∗*p* < 0.01 and ∗∗∗*p* < 0.001. Individual *p* value are listed in Table S6. ATF4, activating transcription factor 4; CAR, constitutive androstane receptor; CHOP, C/EBP homologous protein; hUGT1, humanized *UGT1*; NRF2, nuclear factor erythroid derived 2-related factor 2; PPARα, peroxisome proliferator-activated receptor alpha; PXR, pregnane-X receptor; RT-qPCR, reverse transcription-quantitative PCR; TCS, triclosan; TSB, total serum bilirubin; UGT1A1, UDP-glucuronosyltransferase 1A1.

We had previously demonstrated that long term exposure to TCS in adults resulted in the activation of ER stress through the PERK-ATF4 pathway, which is linked to activation of PPARα (9). When neonatal *hUGT1* mice were orally treated with TCS (50 mg/kg), the treatment resulted in the induction of ATF4 and CHOP in liver (Fig. 5*A*). To examine the association between ATF4 and UGT1A1 expression, 10-day-old *hUGT1/Atf4*^*F/F*^ and *hUGT1/Atf4*^*ΔHep*^ neonatal mice were treated by oral gavage with TCS for 4 consecutive days. RT-qPCR confirmed the absence of ATF4 and RNA-seq data revealed ablation of *Atf4* and its target genes in the liver of *hUGT1/Atf4*^*ΔHep*^ mice (Fig. S5, *A* and *B*). Following TCS treatment, both *hUGT1/Atf4*^*F/F*^ and *hUGT1/Atf4*^*ΔHep*^ mice showed lower TSB levels when compared to vehicle-treated mice (Fig. 5*B*). However, the reduction of bilirubin levels in *hUGT1/Atf4*^*ΔHep*^ was far more dramatic than *hUGT1/Atf4*^*F/F*^. This finding resulted from dramatic superinduction of UGT1A1 in the TCS-treated *hUGT1/Atf4*^*ΔHep*^ neonates (Fig. 5, *C* and *D*). In addition, we examined gene expression of the other human *UGT1* genes. The human *UGT1* locus in *hUGT1* mice encodes nine unique genes (*UGT1A1*, *1A3*, *1A4*, *1A5*, *1A6*, *1A7*, *1A8*, *1A9*, and *1A10*). There is minimal expression of these genes in neonatal liver, but *UGT1A1*, *UGT1A3*, *UGT1A4*, *UGT1A6*, and *UGT1A9* are expressed constitutively in adult liver. In TCS-treated *hUGT1/Atf4*^*ΔHep*^ neonatal mice, significant superinduction of *UGT1A1*, *UGT1A3*, and *UGT1A4* occurred, with no detectable induction of *UGT1A6* or *UGT1A9* (Fig. 5*C*). Average expression of RNA-seq data showed that *hUGT1/Atf4*^*ΔHep*^ presented increased Nrf2 target genes when compared to *hUGT1/Atf4*^*F/F*^ when both were treated with TCS. On the contrary, the average expression of PPARα, CAR, and PXR target genes were similar between both strains treated with TCS (Fig. 5*E*). We validated these findings with RT-qPCR (Fig. S5, *C* and *D*). Our findings indicate that while TCS may activate PPARα as the driving force in the induction of hepatic UGT1A1, the absence of ATF4 is leading to an increase in ROS production and consequently superinduction of UGT1A1, UGT1A3, and UGT1A4 in neonatal *hUGT1* mice. This finding may also explain why the levels of UGT1A1 in neonates are developmentally delayed.

## Discussion

Long-term exposure to TCS has been linked to several metabolic diseases, including HCC and NAFLD (7, 8, 9). In addition, chronic exposure to TCS leads to ulcerative colitis, which has recently been linked to reactivation of TCS from TCS-G by specific intestinal microbial β-glucuronidase enzymes (5). Exposure of newborns to TCS may be more harmful than to adults since hepatic gene expression of *UGT1A1* and other human *UGT1A* genes are repressed shortly after birth and during the neonatal period (38). In addition, metabolic profiles in newborn liver are dramatically different from that of adults (39). Epidemiology findings have confirmed the presence of TCS in human breastmilk (6, 40) and recent findings showed that lactational delivery of TCS is linked to pediatric steatotic liver disease in newborn mice (9). In addition, lactational TCS exposure changes bacterial diversity and decreases microbiota alpha diversity in the human infant gut (40). While an understanding of the impact of TCS on neonatal gene expression and metabolism is limited, findings presented in this report revealed that liver ATF4 and PPARα are important players in UGT1A1 induction.

The actions of TCS on the processes leading to induction of UGT1A1 were initially investigated through identification of NR target genes that were induced following exposure. The human *UGT1A1* gene has been characterized to be responsive to a host of NRs, including LXRα, PXR, CAR, PPARα, and the AhR (14, 16, 18, 19, 21). While there is mild induction of PXR target genes, the most significant response was induction of the *Cyp2b10* and *Cyp4a10* genes, driven by activated CAR and PPARα, respectively. The deletion of CAR in *hUGT1/Car*^*−/−*^ led to the elimination of TCS-induced hepatic *Cyp2b10* gene expression, while PPARα deletion in *hUGT1/Pparα*^*−/−*^ mice resulted in elimination of induced *Cyp4a10* gene expression. Since activated CAR is known to induce human *UGT1A1* gene expression, we fully expected TCS exposure to neonatal *hUGT1* mice to lead to hepatic *UGT1A1* gene expression. Yet, in both neonatal and adult *hUGT1* mice, TCS exposure led to the induction of the *Cyp2b10* gene yet had no impact on inducing UGT1A1. Although TCS activates CAR, we can speculate that additional cofactors are needed to form a functional CAR-specific transcriptional unit for the induction of the *UGT1A1* gene that are not needed for induction of the *Cyp2b10* gene.

We had previously demonstrated that lactational delivery of TCS promotes NAFLD in newborn mice through the activation of ER stress which engages the PERK-eIF2α-ATF4-PPARα cascade (9). Additionally, TCS is unable to stimulate PPARα-directed transcriptional activation of reporter gene constructs driven by PPARα enhancer elements transfected into CV-1 cells. Thus, TCS is not a direct ligand for PPARα (7). However, the activation of PPARα by Pirinixic acid (WY-14643), a potent PPARα ligand, can induce UGT1A1, UGT1A3, UGT1A4, and UGT1A6 in the liver of *hUGT1* mice (21) in a fashion that resembles induction of these genes by TCS. In addition, activation of PPARα by TCS in liver leads to fatty acid metabolism, lipogenesis, and ketogenesis. When we treated *Pparα*^*−/−*^ mice with TCS, these liver functions were abrogated (9). When *hUGT1* mice were treated with TCS, hepatic *Cyp4a10* was significantly induced, indicating that oral TCS does lead to the activation of PPARα. To understand if PPARα activation by TCS underlies induction of hepatic UGT1A1, we treated both *hUGT1* and *hUGT1/Pparα*^*−/−*^ with TCS. In the absence of PPARα, there was no expression of hepatic UGT1A1 following TCS exposure. With TCS not serving as a direct PPARα ligand, we propose that its activation is advanced by activation of ER stress and the PERK-elF2α-ATF4-PPARα cascade (9).

To investigate this possibility further, we examined the actions of ATF4 in the induction of UGT1A1. Induction of ATF4 by TCS in mice on a high-fat diet leads to fat accumulation in hepatic tissue (8). In adult mice, when we targeted the deletion of ATF4 in *Atf4*^*ΔHep*^ mice, the high-fat diet combined with TCS treatment did not accumulate hepatic fatty acids (8). Free fatty acids are PPARα endogenous ligands. In our experiments, 10-day-old neonatal mice are still nursing through lactation and receiving a high-fat diet through breast milk. Thus, treatment of 10-day-old neonatal mice with TCS simulates experiments conducted in adults who are on a high-fat diet. Since ATF4 is induced in concordance with induction of hepatic UGT1A1 in 10-day-old *hUGT1* neonatal mice, we examined the role of ATF4 in the induction process. Following TCS treatment, UGT1A1 was induced in liver tissue from *hUGT1/Atf4*^*F/F*^ mice. However, when we treated 10-day-old *hUGT1/Atf4*^*ΔHep*^ mice with TCS, there was a dramatic induction of liver *UGT1A1* gene expression that could be reflected as superinduction of UGT1A1. Since the induction of UGT1A1 in *hUGT1* mice is far less than observed in *hUGT1/Atf4*^*F/F*^ mice, it can be interpreted that ATF4 is functioning in part to suppress the underlying mechanisms leading to TCS induction of liver UGT1A1.

In the absence of hepatic ATF4 (targeted liver knockout) and a rich diet of fats and lipids coming from breast milk, lipid accumulation in the liver following TCS treatment would be dramatically reduced, which we have previously demonstrated (9). Since ATF4 is a critical player in the PERK-elF2α-ATF4-PPARα pathway, the reduction in hepatic lipid accumulation would not be expected to activate PPARα leading to superinduction. However, activation of PPARα following TCS treatment is central to the induction of hepatic UGT1A1, since if it is deleted, there is no induction of UGT1A1. It is also worth noting that TCS induces UGT1A1 in both neonates and adult mice, but superinduction in *hUGT1/Atf4*^*ΔHep*^ mice only occurs in neonates (Fig. S5*E*). In addition, ATF4-driven superinduction is selective and not necessarily linked to all PPARα target genes. PPARα target genes *Cyp4a10*, *Cyp4a14*, and *Ehhadh* were slightly reduced in *hUGT1/Atf4*^*ΔHep*^ vehicle mice, yet the administration of TCS only led to induction of these genes in a pattern that was comparable to induction in *hUGT1* mice. The deletion of ATF4 followed by TCS exposure did not result in superinduction of these genes. Along with the *UGT1A1* gene, superinduction in *hUGT1/Atf4*^*ΔHep*^ mice also occurred with other *UGT1A* genes, including UGT1A3 and UGT1A4. The other *UGT1A* genes expressed in liver tissue, that included UGT1A6 and UGT1A9, did not result in superinduction. RNA-seq analysis revealed that ATF4 has a protective role in the liver when mice are exposed to TCS. Previous studies demonstrated that deletion of ATF4 and exposure to TCS increases the expression of 4-hydroxynonenal and malondialdehyde, biomarkers of lipid peroxidation (33, 41). 4-hydroxynonenal is considered to be one of the major generators of ROS and oxidative stress (42). Consequently, an increase in ROS production and oxidative stress leads to an activation of Nrf2, which is the major transcriptional regulator of redox homeostasis (35). Of note, previous studies in our laboratory demonstrated that the generation of ROS caused by arsenic and isothiocyanates leads to the activation of Nrf2 and consequently induction of UGT1A1 (23, 24).

Lactational and oral exposure of neonatal *hUGT1* mice to TCS has a significant impact on NRs and transcriptional factors in the liver. We have confirmed that UGT1A1 induction by TCS is exclusively in the liver, and PPARα is a key NR involved in this process. We proposed in previous studies the presence of an ATF4-PPARα axis which is activated following TCS exposure (8, 9). Based upon our findings, ATF4 also plays an important role in the induction of UGT1A1. The deletion of ATF4 in the liver increases ROS production in the liver of mice exposed to TCS. The generation of ROS activates the target genes of activated Nrf2. The superinduction of UGT1A1 that we observed in *hUGT1/Atf4*^*ΔHep*^ suggests that ATF4 protects the liver from excessive oxidative stress in the neonatal stage. Superinduction also provides evidence that the expression of ATF4 in neonatal *hUGT1* hepatic tissue may play an important role in the developmental repression of UGT1A1.

## Experimental procedures

### Animals and treatment

Transgenic mice expressing the human *UGT1* locus in a *Ugt1*^*−/−*^ background (*hUGT1*) were developed previously (15). The Car-null (*Car*^−/−^) mice was a generous gift from Masahiko Negishi (National Institute of Environmental Health Sciences). *hUGT1/Car*^*−/−*^ mice were constructed crossing *hUGT1* with *Car*^*−/−*^. The *Pparα*-null (*Pparα*^*−/−*^) mice were purchased from Jackson Laboratory, and *hUGT1/Pparα*^*−/−*^ mice were constructed crossing *hUGT1* with *Pparα*^−/−^ mice. *Atf4*^F/F^ mice were a generous gift from Dr Christopher M. Adams (University of Iowa). These mice were intercrossed with *Alb-Cre* mice to generate *Atf4*^F/F^ and *Atf4*^*ΔHep*^ mice as previously described (9). *Atf4*^*ΔHep*^ mice were crossbred with *hUGT1* mice to obtain *hUGT1/Atf4*^*F/F*^ and *hUGT1/Atf4*^*ΔHep*^ mice. All mouse lines were in the C57BL/6 genetic background and housed in a pathogen-free UCSD Animal Care Facility and received food and water *ad libitum*. The protocols for mouse handling and procedures were approved by the UCSD Animal Care (S99100) and Use Committee (IACUC), and these protocols were conducted in accordance with federal regulations. In neonatal studies, male and female pups at 10 days old with body weight between 6.0 g and 8.0 g were used. Each experimental finding was obtained from at least two different litters. In each litter, mice were randomly divided into control and treated groups. Littermate controls were used for all experiments. The sample size calculation was based on serum TSB levels from vehicle and treated mice. We have employed the website (https://clincalc.com/stats/samplesize.aspx) for sample size calculations. The power of the experiment was set to 90%, and the calculated N value for each group is 3. Therefore, we used at least three mice in each group in the following experiments.

For breastfeeding studies, male and female mating *hUGT1* mice were fed normal chow with 120 ppm (0.012%) TCS (Sigma-Aldrich, 72779) dissolved in DMSO (10%) and water (90%). Vehicle groups received only DMSO in water (9). This diet was continued after birth and for the next 14 days, at which time vehicle and TCS breastfed newborn mice were sacrificed, and their livers, small intestine, and blood were collected for further analysis. Blood bilirubin levels and tissues, gene expression, and protein levels were quantitated. To analyze the delivery of TCS by lactation, we performed a foster study. Seven-day-old neonatal mice born from control females were transferred to a cage with nurturing females fed with TCS (Veh foster). Also, 7-day-old neonatal mice born from TCS breastfed mice were transferred to a cage where the mice were not receiving TCS (TCS foster).

For dose-dependent studies in neonates, 10-day-old *hUGT1* mice were treated by oral gavage with vehicle or 25, 50, and 100 mg/kg TCS for 4 consecutive days. In studies using *hUGT1*/*Pparα*^*−/−*^, *hUGT1/Car*^*−/−*^, and *hUGT1/Atf4*^*ΔHep*^ mice, 10-day-old mice were treated by oral gavage with vehicle or 50 mg/kg TCS, for 4 consecutive days. After treatment, neonatal mice were sacrificed, and blood, livers, and small intestines were collected and subjected to further analysis.

For adult treatment, 6-weeks-old hUGT1, *hUGT1*/*Pparα*^*−/−*^, and *hUGT1/Atf4*^*ΔHep*^ mice were treated by oral gavage with vehicle or 50 mg/kg TCS, for 4 consecutive days. After treatment, adult mice were sacrificed, and blood and livers were collected and subjected to further analysis.

### Bilirubin measurements

Serum was collected from the mandibular vein in neonates, allowed to clot in an Eppendorf tube, and centrifuged at 14,000*g* for 2 min. The serum samples were used to measure bilirubin levels (mg/dl) using a Unistat Bilirubinometer (Reichert, Inc).

### Serum TCS by liquid spectrometry (HPLC)

To each sample, 20 μl of serum was transferred to a clean 1.5 ml microfuge tube. To each sample, 80 μl of ethanol extraction solvent containing 250 nM of ^13^C-labeled TCS was added. Samples were then vortexed at 2000 rpm for 5 min at 4 °C to allow for protein precipitation followed by centrifugation at 14,000 rpm for 5 min at 4 °C. For each sample, 75 μl of supernatant was transferred to an amber glass HPLC vial (P/N 92-5182-0716) containing a Wheaton 300 μl (P/N 11-0000-100) glass insert. Samples were stored at 4 °C in a Thermo Scientific Vanquish UHPLC autosampler until analysis by LC-MS/MS. Samples were processed and analyzed as previously described (9).

### RT-qPCR and RNA sequencing

Tissue samples were homogenized in 1 ml TRIzol Reagent (Thermo Fisher Scientific, 15596026) and total RNA was isolated. Using iScript Reverse Transcriptase (Bio-Rad, 1708891), 1 μg of total RNA was used for the generation of cDNA in a total volume of 8 μl as outlined by the manufacturer. Following cDNA synthesis, quantitative PCR was carried out on a CFX96 qPCR system by using Ssoadvanced SYBR Green reagent (Bio-Rad, 1725274). All primers used were purchased from Integrated DNA technology and the sequences are in Table S1.

For RNA-sequencing, 14-day-old *h*UGT1/*Atf4*^*F/F*^ and *hUGT1/Atf4*^*ΔHep*^ mice were treated with vehicle or TCS (50 mg/kg for 4 consecutive days) and the liver samples were collected for total RNA isolation. The extracted RNA samples from two mice were combined to represent a single sample. For four groups, RNA-seq analysis was run on two samples per group (four mice for each treatment). The sequencing library for each sample was prepared and analyzed as previously described (24).

### Western blot analysis

Liver tissue (0.1 mg) was homogenized in 0.4 ml 1 X RIPA lysis buffer (EMD Millipore, 20-188) supplemented with protease and phosphatase inhibitors (Thermo Fisher Scientific, 87786 and 78420). For cell fractionation analysis, 0.4 g minced liver tissue were homogenized in 0.5 ml buffer A (10 mM Hepes, 1.5 Mm MgCl_2_, 10 mM KCl, and 0.5 mM DTT, and 0.05% Igepal, pH 7.9) supplemented a with protease and phosphatase inhibitor cocktail. After homogenization, liver homogenates were centrifuged at 3500*g* for 10 min at 4 °C, and the supernatants (cytosolic fraction) were transferred to a new tube and kept at −80 °C until further analysis. For isolated nuclear fractions, the pellets were resuspended in 0.2 ml of buffer B (5 Mm Hepes, 1.5 Mm MgCl_2_, 0.2 mM EDTA, 0.5 mM DTT, and 26% glycerol, pH 7.9) supplemented with 300 mM NaCl, and each sample was sonicated three times. After sonication, the samples were centrifuged 15,000*g* for 20 min at 4 °C, and the supernatants (nuclear fraction) transferred to a new tube and kept at −80 °C until further analysis.

Western blot analysis was performed using NuPAGE 4 to 12% BisTris-polyacrylamide gels (Thermo Fisher Scientific, NW04127BOX) with the protocols described by the manufacturer. Protein (30 μg) was subject to electrophoresis at 170 V for 50 min and the proteins transferred at 20 V for 2 h to PVDF membranes (EMD Millipore, IPVH00010). Membranes were blocked with 5% non-fat milk at room temperature for 1 h and incubated with primary antibodies, at 4 °C overnight. Primary antibodies used for Western Blotting were rabbit anti-UGT1A1 (Abcam, ab170858), rabbit anti-ATF4 (Cell Signaling, CS11815), mouse anti-GAPDH (Santa Cruz Technologies, sc-32233), rabbit anti-CHOP (Cell Signaling, CS5554), rabbit anti-CAR (Abclonal, A1970), and rabbit anti-CYP2B10 (a kind gift from Dr Masahiko Negishi, NIEHS). Membranes were washed and exposed to HRP-conjugated secondary antibodies (anti-mouse IgG, anti-rabbit IgG and anti-goat, Cell Signaling Technology) for 1 h at room temperature. All primary antibodies were diluted 1:1000, and all secondary antibodies were diluted 1:3000. Protein was detected by the ECL Plus Western blotting detection system and was visualized by the Bio-Rad ChemiDoc Touch Imaging System. All Western blot pictures were cropped by using Image Lab 5.2.1 software. The relative expression of all Western blot bands is reported in Tables S11–S17.

### Histology and immunohistochemistry

To analyze liver morphology, tissue samples were fixed in 10% buffered formalin phosphate (Fisher Chemicals, SF100-4), transferred to 70% ethanol, and processed at the UCSD Tissue Technology. Samples were embedded in paraffin, sliced into 5 μm sections, and stained with H&E (hematoxylin and eosin). For the staining of UGT1A proteins (Santa Cruz Technologies, sc-271268), paraffin liver sections were prepared in the UCSD Tissue Technology. Formalin-fixed, paraffin-embedded liver slides were deparaffinized and rehydrated, using xylene followed by alcohol and PBS washings. Antigen retrieval of tissue slides and the immunohistochemical staining with a primary antibody, secondary biotinylated antibody (BD Pharmingen, 550337), and Avidin D (Vector Laboratories, A-2004) were achieved as described previously (7). Primary antibody was diluted 1:100, and secondary antibody was diluted 1:200. The images were captured on an upright light/fluorescent microscope (Zeiss) equipped with AxioCam camera.

### Statistical analyses

Data are represented as mean ± standard deviation (SD). For all data, we used Shapiro–Wilk test to verify the normality of data; when data are normally distributed, statistical significance was determined using unpaired two-sided Student’s *t* test, otherwise, significance was determined by Wilcoxon–Mann–Whitney test. *p* values <0.05 were considered statistically significant, and statistically significant differences are indicated with ∗*p* < 0.5; ∗∗*p* < 0.01; ∗∗∗*p* < 0.001, and ∗∗∗∗*p* < 0.0001. Statistical analyses were performed using GraphPad 9.1.0. The results of all statistical comparisons between groups and number of samples are indicated in each figure with exact *p* values shown in Tables S2–S10.

## Data availability

The data reported in this paper have been deposited in the Gene Expression Omnibus database with the accession code GSE264293. All other data generated or analyzed during this study are available from the corresponding author on reasonable request.

## Supporting information

This article contains supporting information.

## Conflict of interest

The authors declare that they have no conflicts of interest with the contents of this article.
