# Supplementary material for: Triclosan administration to humanized UDP-glucuronosyltransferase 1 neonatal mice induces UGT1A1 through a dependence on PPARα and ATF4
Source: J Biol Chem. 2024 May 4;300(6):107340. doi: 10.1016/j.jbc.2024.107340 (PMC11152660; doi:10.1016/j.jbc.2024.107340)
Supplement: Supplemental Figures S1–S5 and Tables S1–S17 [file mmc1.docx]

**SUPPORTING INFORMATION**

**Triclosan administration to *humanized UDP-glucuronosyltransferase1* neonatal mice induces UGT1A1 through a dependence on PPARα and ATF4**

André A. Weber^1^, Xiaojing Yang^1^, Elvira Mennillo^1^, Samantha Wong^1^, Sabrina Le^1^, Jia Ying Ashley Teo^1^, Max Chang^2^, Christopher Benner^2^, Jeffrey Ding^2^, Mohit Jain^2^, Shujuan Chen^1^, Michael Karin^3^, Robert H. Tukey^1^.

^1^Laboratory of Environmental Toxicology, Department of Pharmacology, University of California, San Diego, La Jolla, CA, USA.

^2^Department of Medicine, School of Medicine, University of California, San Diego, La Jolla, CA, USA.

^3^Laboratory of Gene Regulation and Signal Transduction, Department of Pharmacology, School of Medicine, University of California San Diego, La Jolla, CA, USA.

Address correspondence to: Robert H. Tukey, Department of Pharmacology, University of California, San Diego 92093-0722. E-mail: [rtukey@health.ucsd.edu](mailto:rtukey@health.ucsd.edu)

**Running Title**: PPARα and ATF4 regulate glucuronidation of TCS in neonates.

**
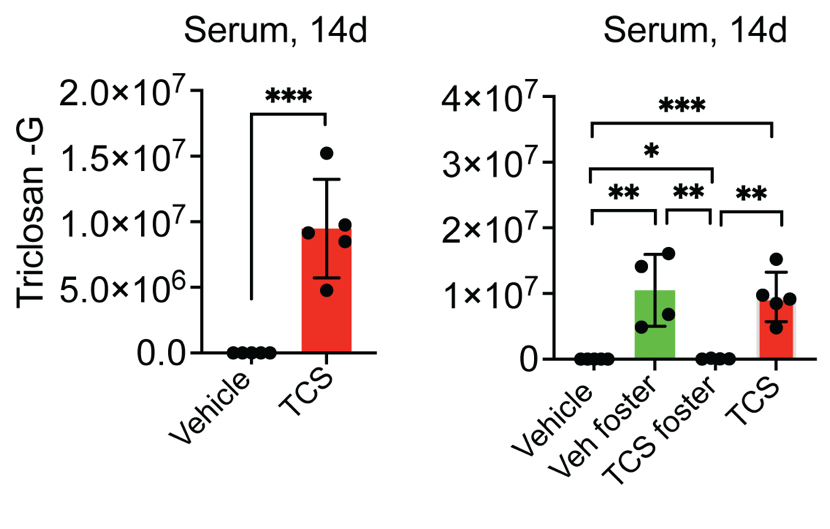
**

**Supporting Figure 1. TCS glucuronide (TCS-G) levels.** TCS-G levels in neonatal mice treated through lactation (n=5) and foster experiment (vehicle and TCS, n=5 and veh foster and TCS foster, n=4). Results are described as mean ± SD. *p<0.05, **p<0.01, ***p<0.001, Student’s t test. Individual P value was listed in supporting information table S7.


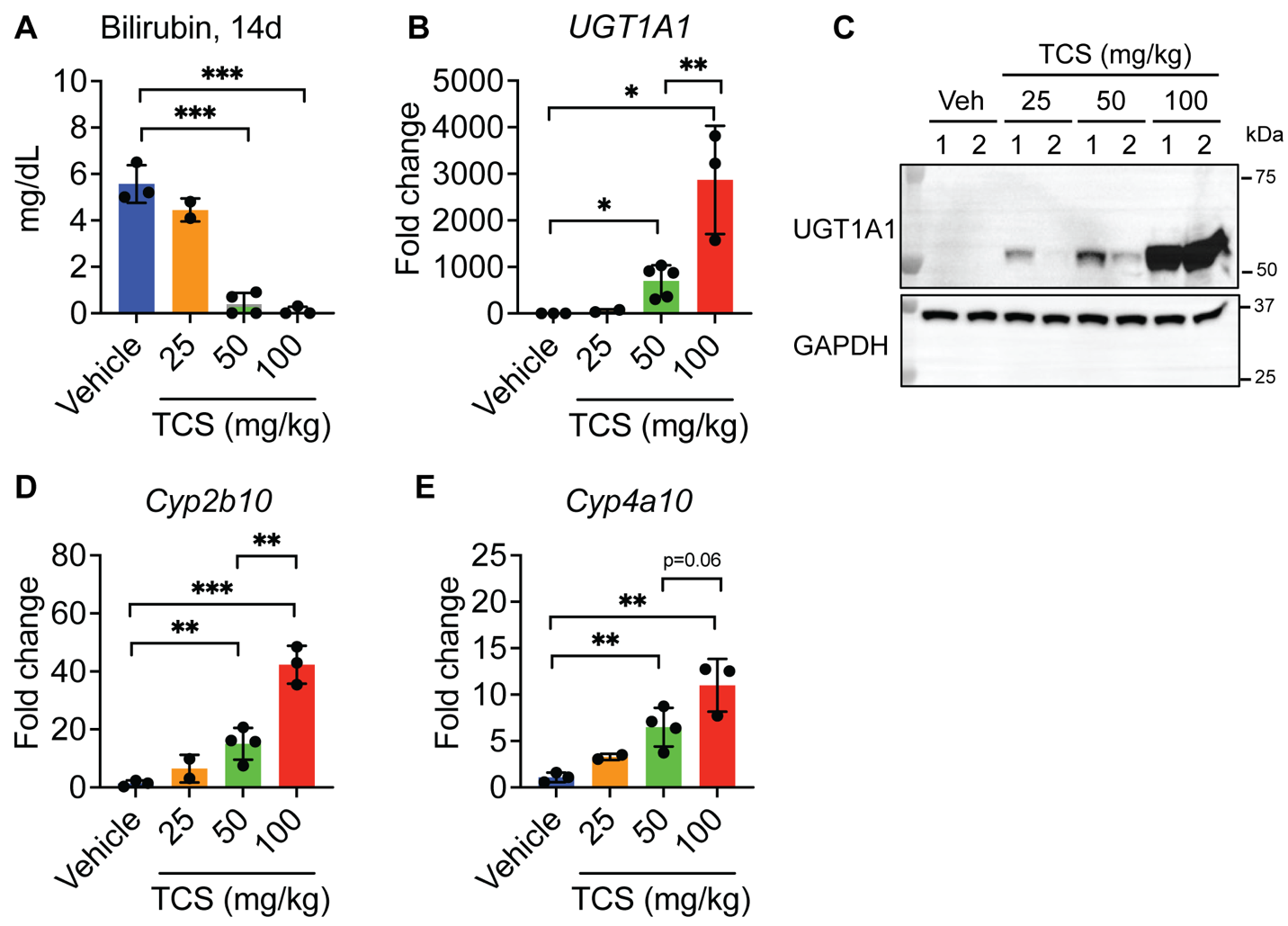


**Supporting Figure 2. Dose dependent TCS oral gavage studies**. 10 days-old *hUGT1* mice were treated with a range of TCS concentrations (25, 50 and 100 mg/kg) for four consecutive days. After treatment, the neonates were sacrificed, and serum and liver were collected for analysis. (**A**) TSB levels (n=3, 2, 4, 3). (**B**) Fold change of hepatic *UGT1A1* expression (n=3, 2, 5, 3). (**C**) Western blot analysis of UGT1A1 in the liver. GAPDH was used as loading control (n=2 per group). (**D**) Fold change of hepatic *Cyp2b10* expression (n=3, 2, 4, 3). (**E**) Fold change of hepatic *Cyp4a10* expression (n=3, 2, 4, 3). Results are described as mean ± SD. *p<0.05, **p<0.01, ***p<0.001, Student’s t test. Individual P value was listed in supporting information table S8.


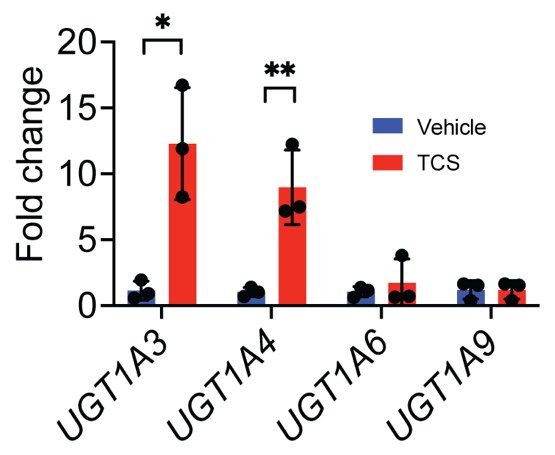


**Supporting Figure 3.** **Hepatic** **human UGT1A isoforms activation by TCS**. 10 days-old *hUGT1* mice were treated with 50 mg/kg TCS for four consecutive days. After treatment, livers were collected for analysis. RT-qPCR analysis of the different *UGT1A* genes (n=3). Results are described as mean ± SD. *p<0.05, **p<0.01, Student’s t test. Individual P value was listed in supporting information table S9.


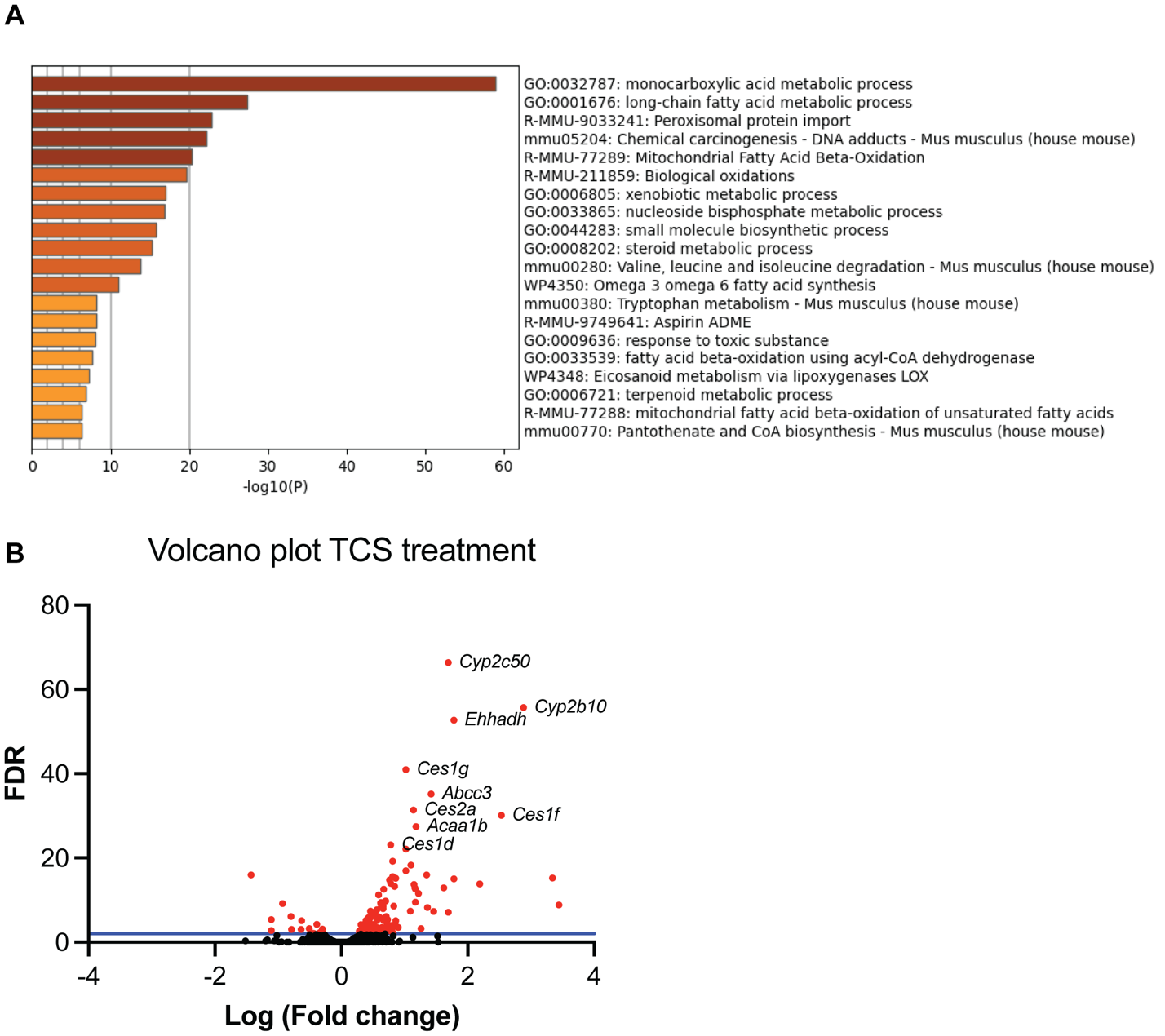


**Supporting Figure 4.** **Oral TCS treatment dramatically affect liver transcriptome**. 10-day old *hUGT1* mice were treated with 50 mg/kg TCS or vehicle for four consecutive days. After treatment, livers were collected for analysis. (**A**) DAVID-based GO analysis revealed the main processes affected by TCS treatment. (**B**) Volcano plot showing genes in red with false discovery rate (FDR) higher than 2, between vehicle and TCS treatment.


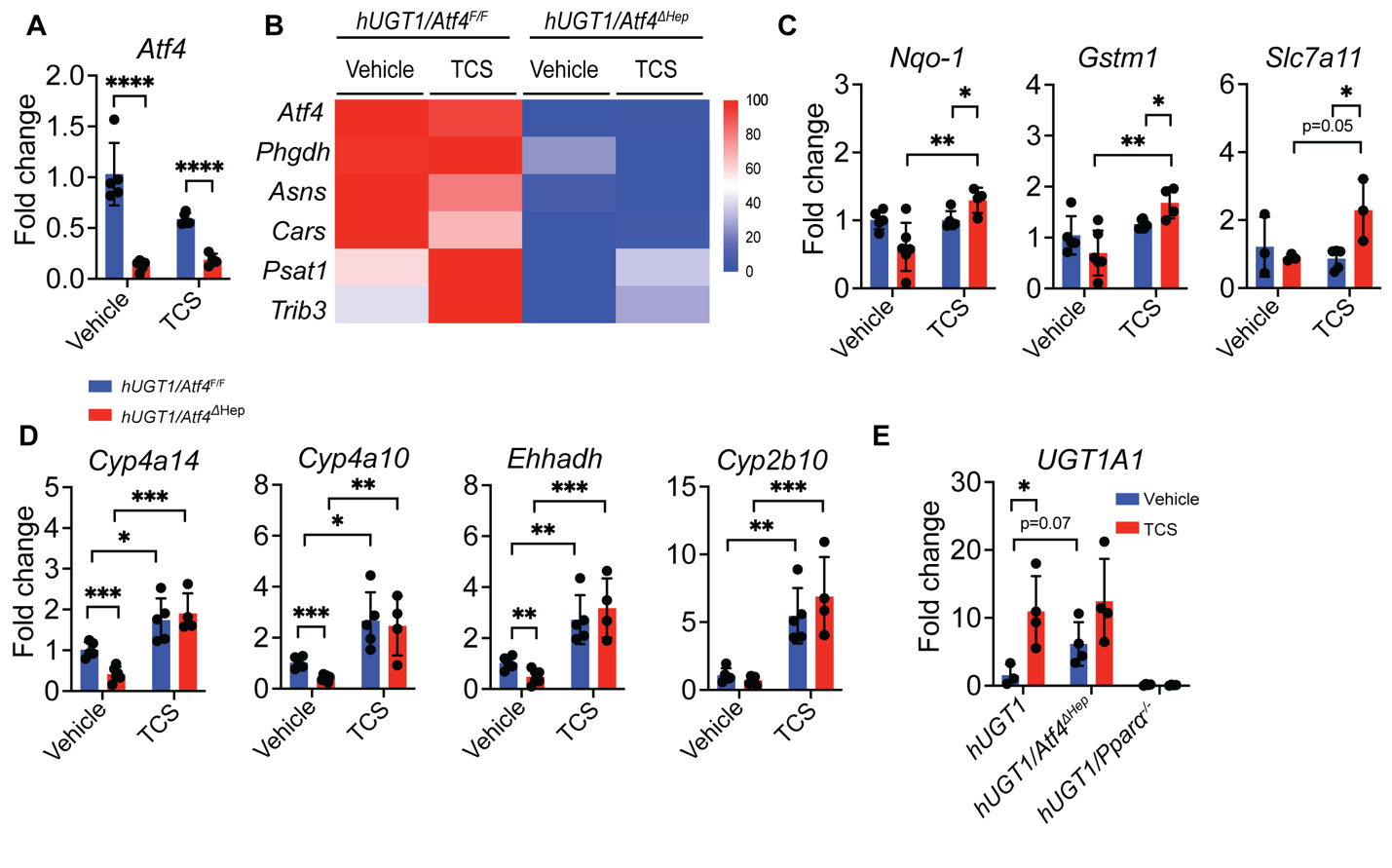


**Supporting Figure 5. The impact of ATF4 on expression of the UGT1A1 and nuclear receptors target genes in liver of neonates and adults.** (**A**) 10 days-old *hUGT1/Atf4^F/F^* and *hUGT1/Atf4^∆Hep^* mice were treated with 50 mg/kg for four consecutive days. After treatment, the neonates were sacrificed, and liver were collected for RT-PCR analysis. Fold change of hepatic *Atf4* (n=5, 6, 5, 4). (**B**) RNA-seq showing different expression of *Atf4* and its targets genes. (**C**) Fold change of hepatic NRF2 target genes, *Nqo-1*, *Gstm1* and *Slc7a11*. (**D**) Fold change of hepatic PPARα target genes, *Cyp4a10*, *Cyp4a14*, *Ehhadh* and CAR target gene, *Cyp2b10* gene expression (n=5, 6, 5, 4). (**E**) 6 weeks-old *h*UGT1, *h*UGT1/*Atf4^∆Hep^* and *hUGT1/Pparα^-/-^* mice were treated with 50 mg/kg for four consecutive days. After treatment, the neonates were sacrificed, and liver were collected for RT-PCR analysis. Fold change of hepatic UGT1A1 (n=3, 4, 4, 4, 4, 4). Results are described as mean ± SD. **p*<0.05, ***p*<0.01, ****p*<0.001 and *****p*<0.0001. Student’s t test. Individual P value was listed in supporting information table S10.

**Supporting Table 1.** Primers used for real-time PCR analysis of selected genes.

| **Gene** | **Forward** | **Reverse** |
| --- | --- | --- |
| *Atf4* | TTGTCCGTTACAGCAACACTG | GCAGCAGCACCAGGCTCT |
| *Cyclophiline* | CAGACGCCACTGTCGCTT | TGTCTTTGGAACTTTGTCTGC |
| *Cyp2b10* | TGCAGATGGACAGAGGAGG | CACACAGCATAACCACAGGC |
| *Cyp3a11* | TTCTGTCTTCACAAACCGGC | GGGGGACAGCAAAGCTCTAT |
| *Cyp4a10* | GATGGACGCTCTTTACCCAA | AAGGGTCAAACACCTCTGGA |
| *Cyp7a1* | GGGAATGCCATTTACTTGGA | GTCCGGATATTCAAGGATGC |
| *Cyp1a1* | TGCCCTTCATTGGTCACATG | CACGTCCCCATACTGCTGACT |
| *Ehhadh* | CTATGATCCGCCTCTGCAA | TGGCTCTAACCGTATGGTCC |
| *Nqo1* | GGTGATATTTCAGTTCCCATTGC | GCAGGATGCCACTCTGAATC |
| *UGT1A1* | AACAAGGAGCTCATGGCCTCC | GTTCGCAAGATTCGATGGTCG |
| *UGT1A3* | TCAACTGTGCCAACAGGAAG | CTGAGACCATTGATCCCAAAG |
| *UGT1A4/5* | CAACGGGAAGCCACTATCTC | TGAGACCATTGATCCCAAAGA |
| *UGT1A6* | AATTTCCTAAAGGCCGGTCA | ACCACAATTCCATGTTCTCCA |
| *UGT1A7* | TGTCATCAGGGAAAGCCAGT | TGAGACCATTGATCCCAAAGA |
| *UGT1A8* | GGTATCAACTGCCATCAGGG | CTGAGACCATTGATCCCAAAG |
| *UGT1A9/10* | TGATGCCCAACATGATCTTC | CCACAATTCCATGTTCTCCA |

**Supporting Table 2.** Data analysis details for **Figure 1**.

| **Figure 1** | **Group** | **Analysis** | **P value** | **P value summary** |
| --- | --- | --- | --- | --- |
| *Fig 1A* | Veh vs TCS | Unpaired t test | <0.0001 | **** |
| *Fig 1A* | Veh vs Veh foster | Unpaired t test | 0.0002 | *** |
| *Fig 1A* | Veh vs TCS foster | Unpaired t test | 0.15 | ns |
| *Fig 1A* | Veh vs TCS | Unpaired t test | <0.0001 | **** |
| *Fig 1A* | Veh foster vs TCS foster | Unpaired t test | 0.006 | ** |
| *Fig 1A* | Veh foster vs TCS | Unpaired t test | 0.74 | ns |
| *Fig 1A* | TCS foster vs TCS | Unpaired t test | 0.0004 | *** |
| *Fig 1B* | Veh vs TCS | Unpaired t test | 0.005 | ** |
| *Fig 1B* | Veh vs Veh foster | Unpaired t test | 0.0016 | ** |
| *Fig 1B* | Veh vs TCS foster | Unpaired t test | 0.1193 | ns |
| *Fig 1B* | Veh vs TCS | Unpaired t test | 0.0033 | ** |
| *Fig 1B* | Veh foster vs TCS foster | Unpaired t test | 0.0003 | *** |
| *Fig 1B* | Veh foster vs TCS | Unpaired t test | 0.0435 | * |
| *Fig 1B* | TCS foster vs TCS | Unpaired t test | 0.0019 | ** |
| *Fig 1C* (Liver) | Veh vs TCS | Unpaired t test | 0.0006 | *** |
| *Fig 1C* (SI) | Veh vs TCS | Unpaired t test | 0.0743 | ns |
| *Fig 1C* | Veh vs Veh foster | Unpaired t test | 0.0416 | * |
| *Fig 1C* | Veh vs TCS foster | Unpaired t test | 0.20 | ns |
| *Fig 1C* | Veh vs TCS | Unpaired t test | 0.0153 | * |
| *Fig 1C* | Veh foster vs TCS foster | Unpaired t test | 0.0611 | ns |
| *Fig 1C* | Veh foster vs TCS | Unpaired t test | 0.0486 | * |
| *Fig 1C* | TCS foster vs TCS | Unpaired t test | 0.0170 | * |

**Supporting Table 3.** Data analysis details for **Figure 2**.

| **Figure 1** | **Group** | **Analysis** | **P value** | **P value summary** |
| --- | --- | --- | --- | --- |
| *Fig 2A* | Veh vs TCS (*Cyp2b10*) | Unpaired t test | 0.0004 | *** |
| *Fig 2A* | Veh vs TCS (*Cyp4a10*) | Unpaired t test | 0.0023 | ** |
| *Fig 2A* | Veh vs TCS (*Cyp3a11*) | Unpaired t test | 0.0128 | * |
| *Fig 2A* | Veh vs TCS (C | Unpaired t test | 0.9352 | ns |
| *Fig 2A* | Veh vs TCS (*Cyp1a1*) | Unpaired t test | 0.6741 | ns |
| *Fig 2A* | Veh vs TCS (*Nqo1*) | Unpaired t test | 0.0129 | * |
| *Fig 2D* | Veh vs Veh foster | Unpaired t test | 0.0002 | *** |
| *Fig 2D* | Veh vs TCS foster | Unpaired t test | 0.1073 | ns |
| *Fig 2D* | Veh vs TCS | Unpaired t test | 0.0096 | ** |
| *Fig 2D* | Veh foster vs TCS foster | Unpaired t test | 0.1094 | ns |
| *Fig 2D* | Veh foster vs TCS | Unpaired t test | 0.1074 | ns |
| *Fig 2D* | TCS foster vs TCS | Unpaired t test | 0.0442 | * |

**Supporting Table 4.** Data analysis details for **Figure 3**.

| **Figure 1** | **Group** | **Analysis** | **P value** | **P value summary** |
| --- | --- | --- | --- | --- |
| *Fig 3A* | Vehicle vs TCS | Unpaired t test | <0.0001 | **** |
| *Fig 3B* | Vehicle vs TCS (Liver) | Unpaired t test | 0.0017 | ** |
| *Fig 3C* | Vehicle vs TCS (SI) | Unpaired t test | 0.1399 | ns |
| *Fig 3E* | Vehicle vs TCS (*Cyp2b10*) | Unpaired t test | 0.0002 | *** |
| *Fig 3E* | Vehicle vs TCS (*Cyp4a10*) | Unpaired t test | 0.0003 | *** |
| *Fig 3E* | Vehicle vs TCS (*Cyp3a11*) | Unpaired t test | <0.0001 | **** |
| *Fig 3E* | Vehicle vs TCS (*Cyp1a1*) | Unpaired t test | 0.5442 | ns |
| *Fig 3E* | Vehicle vs TCS (*Cyp7a1*) | Unpaired t test | 0.4494 | ns |
| *Fig 3E* | Vehicle vs TCS (*Nqo1*) | Unpaired t test | 0.0015 | ** |

**Supporting Table 5.** Data analysis details for **Figure 4**.

| **Figure 4** | **Group** | **Analysis** | **P value** | **P value summary** |
| --- | --- | --- | --- | --- |
| *Fig 4A Bilirubin* | Veh (*hUGT1*) vs Veh (*hUGT1/Car^-/-^*) | Unpaired t test | 0.2623 | ns |
| *Fig 4A Bilirubin* | Veh (*hUGT1*) vs TCS (*hUGT1*) | Unpaired t test | 0.0003 | *** |
| *Fig 4A Bilirubin* | Veh (*hUGT1/Car^-/-^*) vs TCS (*hUGT1/Car^-/-^*) | Unpaired t test | 0.0017 | ** |
| *Fig 4A UGT1A1* | Veh (*hUGT1*) vs Veh (*hUGT1/Car^-/-^*) | Unpaired t test | 0.4877 | ns |
| *Fig 4A UGT1A1* | Veh (*hUGT1*) vs TCS (*hUGT1*) | Unpaired t test | 0.0110 | * |
| *Fig 4A UGT1A1* | Veh (*hUGT1/Car^-/-^*) vs TCS (*hUGT1/Car^-/-^*) | Unpaired t test | 0.0219 | * |
| *Fig 4A Cyp2b10* | Veh (*hUGT1*) vs Veh (*hUGT1/Car^-/-^*) | Unpaired t test | 0.0477 | * |
| *Fig 4A Cyp2b10* | Veh (*hUGT1*) vs TCS (*hUGT1*) | Unpaired t test | 0.0002 | *** |
| *Fig 4A Cyp2b10* | Veh (*hUGT1/Car^-/-^*) vs TCS (*hUGT1/Car^-/-^*) | Unpaired t test | 0.0643 | ns |
| *Fig 4B Bilirubin* | Veh (*hUGT1*) vs Veh (*hUGT1/Pparα^-/-^*) | Unpaired t test | 0.9487 | ns |
| *Fig 4B Bilirubin* | Veh (*hUGT1*) vs TCS (*hUGT1*) | Unpaired t test | <0.0001 | **** |
| *Fig 4B Bilirubin* | Veh (*hUGT1/ Pparα^-/-^*) vs TCS (*hUGT1/ Pparα^-/-^*) | Unpaired t test | 0.0041 | ** |
| *Fig 4B Bilirubin* | TCS(*hUGT1*) vs TCS (*hUGT1/ Pparα^-/-^*) | Unpaired t test | 0.0001 | *** |
| *Fig 4B UGT1A1* | Veh (*hUGT1*) vs Veh (*hUGT1/ Pparα^-/-^*) | Unpaired t test | 0.1284 | ns |
| *Fig 4B UGT1A1* | Veh (*hUGT1*) vs TCS (*hUGT1*) | Unpaired t test | 0.0017 | ** |
| *Fig 4B UGT1A1* | Veh (*hUGT1/ Pparα^-/-^*) vs TCS (*hUGT1/ Pparα^-/-^*) | Unpaired t test | 0.0665 | ns |
| *Fig 4B UGT1A1* | TCS(*hUGT1*) vs TCS (*hUGT1/ Pparα^-/-^*) | Unpaired t test | 0.0017 | ** |
| *Fig 4B Cyp4a10* | Veh (*hUGT1*) vs Veh (*hUGT1/ Pparα^-/-^*) | Unpaired t test | <0.0001 | **** |
| *Fig 4B Cyp4a10* | Veh (*hUGT1*) vs TCS (*hUGT1*) | Unpaired t test | 0.0003 | *** |
| *Fig 4B Cyp4a10* | TCS (*hUGT1*) vs TCS (*hUGT1/Pparα^-/-^*) | Unpaired t test | <0.0001 | **** |

**Supporting Table 6.** Data analysis details for **Figure 5**.

| **Figure 5** | **Group** | **Analysis** | **P value** | **P value summary** |
| --- | --- | --- | --- | --- |
| *Fig. 5B* | *hUGT1/Atf4^F/F^* veh vs *hUGT1/Atf4^ΔHep^* veh | Unpaired t test | 0.62 | ns |
| *Fig. 5B* | *hUGT1/Atf4^F/F^* veh vs *hUGT1/Atf4^F/F^* TCS | Unpaired t test | 0.0002 | *** |
| *Fig. 5B* | *hUGT1/Atf4^ΔHep^* veh vs *hUGT1/Atf4^ΔHep^* TCS | Unpaired t test | 0.0012 | ** |
| *Fig 3B* | *hUGT1/Atf4^F/F^* TCS vs *hUGT1/Atf4^ΔHep^* TCS | Unpaired t test | 0.0265 | * |
| *Fig. 5C (UGT1A1)* | *hUGT1/Atf4^F/F^* veh vs *hUGT1/Atf4^ΔHep^* veh | Unpaired t test | 0.0631 | ns |
| *Fig. 5C (UGT1A1)* | *hUGT1/Atf4^F/F^* veh vs *hUGT1/Atf4^F/F^* TCS | Unpaired t test | 0.0131 | * |
| *Fig. 5C (UGT1A1)* | *hUGT1/Atf4^ΔHep^* veh vs *hUGT1/Atf4^ΔHep^* TCS | Unpaired t test | 0.0062 | ** |
| *Fig. 5C (UGT1A1)* | *hUGT1/Atf4^F/F^* TCS vs *hUGT1/Atf4^ΔHep^* TCS | Unpaired t test | 0.0442 | * |
| *Fig. 5C (UGT1A3)* | *hUGT1/Atf4^F/F^* veh vs *hUGT1/Atf4^ΔHep^* veh | Unpaired t test | 0.9271 | ns |
| *Fig. 5C (UGT1A3)* | *hUGT1/Atf4^F/F^* veh vs *hUGT1/Atf4^F/F^* TCS | Unpaired t test | 0.0311 | * |
| *Fig. 5C (UGT1A3)* | *hUGT1/Atf4^ΔHep^* veh vs *hUGT1/Atf4^ΔHep^* TCS | Unpaired t test | 0.0222 | * |
| *Fig. 5C (UGT1A3)* | *hUGT1/Atf4^F/F^* TCS vs *hUGT1/Atf4^ΔHep^* TCS | Unpaired t test | 0.0552 | ns |
| *Fig. 5C (UGT1A4)* | *hUGT1/Atf4^F/F^* veh vs *hUGT1/Atf4^ΔHep^* veh | Unpaired t test | 0.0833 | ns |
| *Fig. 5C (UGT1A4)* | *hUGT1/Atf4^F/F^* veh vs *hUGT1/Atf4^F/F^* TCS | Unpaired t test | 0.0427 | * |
| *Fig. 5C (UGT1A4)* | *hUGT1/Atf4^ΔHep^* veh vs *hUGT1/Atf4^ΔHep^* TCS | Unpaired t test | 0.027 | * |
| *Fig. 5C (UGT1A4)* | *hUGT1/Atf4^F/F^* TCS vs *hUGT1/Atf4^ΔHep^* TCS | Unpaired t test | 0.0606 | ns |

**Supporting Table 7.** Data analysis details for **Figure S1**.

| **Figure 1** | **Group** | **Analysis** | **P value** | **P value summary** |
| --- | --- | --- | --- | --- |
| *Fig S1* | Veh vs TCS | Unpaired t test | 0.0005 | *** |
| *Fig S1* | Veh vs Veh foster | Unpaired t test | 0.0033 | ** |
| *Fig S1* | Veh vs TCS foster | Unpaired t test | 0.0414 | * |
| *Fig S1* | Veh vs TCS | Unpaired t test | 0.0005 | *** |
| *Fig S1* | Veh foster vs TCS foster | Unpaired t test | 0.0089 | ** |
| *Fig S1* | Veh foster vs TCS | Unpaired t test | 0.7556 | ns |
| *Fig S1* | TCS foster vs TCS | Unpaired t test | 00017 | ** |

**Supporting Table 8.** Data analysis details for **Figure S2**.

| **Figure 1** | **Group** | **Analysis** | **P value** | **P value summary** |
| --- | --- | --- | --- | --- |
| *Fig S2A* | Veh vs 50 | Unpaired t test | 0.0001 | *** |
| *Fig S2A* | Veh vs 100 | Unpaired t test | 0.0003 | *** |
| *Fig S2A* | 50 vs 100 | Unpaired t test | 0.3481 | ns |
| *Fig S2B* | Veh vs 50 | Unpaired t test | 0.0135 | * |
| *Fig S2B* | Veh vs 100 | Unpaired t test | 0.0129 | * |
| *Fig S2B* | 50 vs 100 | Unpaired t test | 0.0063 | ** |
| *Fig S2C* | Veh vs 50 | Unpaired t test | 0.0088 | ** |
| *Fig S2C* | Veh vs 100 | Unpaired t test | 0.0004 | *** |
| *Fig S2C* | 50 vs 100 | Unpaired t test | 0.0018 | ** |
| *Fig S2D* | Veh vs 50 | Unpaired t test | 0.0077 | ** |
| *Fig S2D* | Veh vs 100 | Unpaired t test | 0.0040 | ** |
| *Fig S2D* | 50 vs 100 | Unpaired t test | 0.058 | ns |

**Supporting Table 9.** Data analysis details for **Figure S3**.

| **Figure 1** | **Group** | **Analysis** | **P value** | **P value summary** |
| --- | --- | --- | --- | --- |
| *Fig S3A (UGT1A3)* | Veh vs TCS | Unpaired t test | 0.0110 | * |
| *Fig S3A (UGT1A4/5)* | Veh vs TCS | Unpaired t test | 0.0086 | ** |
| *Fig S3A (UGT1A6)* | Veh vs TCS | Unpaired t test | 0.56 | ns |
| *Fig S3A (UGT1A7)* | Veh vs TCS | Unpaired t test | 0.3226 | ns |
| *Fig S3A (UGT1A8)* | Veh vs TCS | Unpaired t test | 0.3648 | ns |
| *Fig S3A (UGT1A9/10)* | Veh vs TCS | Unpaired t test | 0.56 | ns |

**Supporting Table 10.** Data analysis details for **Figure S5**.

| **Figure 1** | **Group** | **Analysis** | **P value** | **P value summary** |
| --- | --- | --- | --- | --- |
| *Fig S5A* | Veh *hUGT1/Atf4^F/F^* and Veh *hUGT1/Atf4^∆Hep^* | Unpaired t test | <0.0001 | **** |
| *Fig S5A* | TCS *hUGT1/Atf4^F/F^* and TCS *hUGT1/Atf4^∆Hep^* | Unpaired t test | <0.0001 | **** |
| *Fig. S5C (Nqo-1)* | *hUGT1/Atf4^F/F^* veh vs *hUGT1/Atf4^ΔHep^* veh | Unpaired t test | 0.0436 | * |
| *Fig. S5C (Nqo-1)* | *hUGT1/Atf4^F/F^* veh vs *hUGT1/Atf4^F/F^* TCS | Unpaired t test | 0.9672 | ns |
| *Fig. S5C (Nqo-1)* | *hUGT1/Atf4^ΔHep^* veh vs *hUGT1/Atf4^ΔHep^* TCS | Unpaired t test | 0.008 | ** |
| *Fig. S5C (Nqo-1)* | *hUGT1/Atf4^F/F^* TCS vs *hUGT1/Atf4^ΔHep^* TCS | Unpaired t test | 0.0287 | * |
| *Fig. S5C (Gstm1)* | *hUGT1/Atf4^F/F^* veh vs *hUGT1/Atf4^ΔHep^* veh | Unpaired t test | 0.1990 | ns |
| *Fig. S5C (Gstm1)* | *hUGT1/Atf4^F/F^* veh vs *hUGT1/Atf4^F/F^* TCS | Unpaired t test | 0.2415 | ns |
| *Fig. S5C (Gstm1)* | *hUGT1/Atf4^ΔHep^* veh vs *hUGT1/Atf4^ΔHep^* TCS | Unpaired t test | 0.0049 | ** |
| *Fig. S5C (Gstm1)* | *hUGT1/Atf4^F/F^* TCS vs *hUGT1/Atf4^ΔHep^* TCS | Unpaired t test | 0.0223 | * |
| *Fig. S5C (Slc7a11)* | *hUGT1/Atf4^F/F^* veh vs *hUGT1/Atf4^ΔHep^* veh | Unpaired t test | 0.5639 | ns |
| *Fig. S5C (Slc7a11)* | *hUGT1/Atf4^F/F^* veh vs *hUGT1/Atf4^F/F^* TCS | Unpaired t test | 0.4284 | ns |
| *Fig. S5C (Slc7a11)* | *hUGT1/Atf4^ΔHep^* veh vs *hUGT1/Atf4^ΔHep^* TCS | Unpaired t test | 0.0579 | ns |
| *Fig. S5C (Slc7a11)* | *hUGT1/Atf4^F/F^* TCS vs *hUGT1/Atf4^ΔHep^* TCS | Unpaired t test | 0.0153 | * |
| *Fig. S5D (Cyp4a14)* | *hUGT1/Atf4^F/F^* veh vs *hUGT1/Atf4^ΔHep^* veh | Unpaired t test | 0.0004 | *** |
| *Fig. S5D (Cyp4a14)* | *hUGT1/Atf4^F/F^* veh vs *hUGT1/Atf4^F/F^* TCS | Unpaired t test | 0.0203 | * |
| *Fig. S5D (Cyp4a14)* | *hUGT1/Atf4^ΔHep^* veh vs *hUGT1/Atf4^ΔHep^* TCS | Unpaired t test | 0.0001 | *** |
| *Fig. S5D (Cyp4a14)* | *hUGT1/Atf4^F/F^* TCS vs *hUGT1/Atf4^ΔHep^* TCS | Unpaired t test | 0.6491 | ns |
| *Fig. S5D (Cyp4a10)* | *hUGT1/Atf4^F/F^* veh vs *hUGT1/Atf4^ΔHep^* veh | Unpaired t test | 0.0003 | *** |
| *Fig. S5D (Cyp4a10)* | *hUGT1/Atf4^F/F^* veh vs *hUGT1/Atf4^F/F^* TCS | Unpaired t test | 0.0124 | * |
| *Fig. S5D (Cyp4a10)* | *hUGT1/Atf4^ΔHep^* veh vs *hUGT1/Atf4^ΔHep^* TCS | Unpaired t test | 0.0022 | ** |
| *Fig. S5D (Cyp4a10)* | *hUGT1/Atf4^F/F^* TCS vs *hUGT1/Atf4^ΔHep^* TCS | Unpaired t test | 0.8044 | ns |
| *Fig. S5D (Ehhadh)* | *hUGT1/Atf4^F/F^* veh vs *hUGT1/Atf4^ΔHep^* veh | Unpaired t test | 0.0085 | ** |
| *Fig. S5D (Ehhadh)* | *hUGT1/Atf4^F/F^* veh vs *hUGT1/Atf4^F/F^* TCS | Unpaired t test | 0.0049 | ** |
| *Fig. S5D (Ehhadh)* | *hUGT1/Atf4^ΔHep^* veh vs *hUGT1/Atf4^ΔHep^* TCS | Unpaired t test | 0.0005 | *** |
| *Fig. S5D (Ehhadh)* | *hUGT1/Atf4^F/F^* TCS vs *hUGT1/Atf4^ΔHep^* TCS | Unpaired t test | 0.5450 | ns |
| *Fig. S5D (Cyp2b10)* | *hUGT1/Atf4^F/F^* veh vs *hUGT1/Atf4^ΔHep^* veh | Unpaired t test | 0.2080 | ns |
| *Fig. S5D (Cyp2b10)* | *hUGT1/Atf4^F/F^* veh vs *hUGT1/Atf4^F/F^* TCS | Unpaired t test | 0.0017 | ** |
| *Fig. S5D (Cyp2b10)* | *hUGT1/Atf4^ΔHep^* veh vs *hUGT1/Atf4^ΔHep^* TCS | Unpaired t test | 0.002 | ** |
| *Fig. S5D (Cyp2b10)* | *hUGT1/Atf4^F/F^* TCS vs *hUGT1/Atf4^ΔHep^* TCS | Unpaired t test | 0.4171 | ns |
| *Fig S5E* | Veh *h*UGT1 vs TCS *h*UGT1 | Unpaired t test | 0.0318 | * |
| *Fig S5E* | Veh *h*UGT1/*Atf4^∆Hep^* vs TCS *h*UGT1/*Atf4^∆Hep^* | Unpaired t test | 0.1228 | ns |
| *Fig S5E* | Veh *hUGT1/Pparα* vs TCS *hUGT1/Pparα* | Unpaired t test | 0.3958 | ns |
| *Fig S5E* | Veh *h*UGT1 vs Veh *h*UGT1/*Atf4^∆Hep^* | Unpaired t test | 0.075 | ns |

**Supporting Table 11.** Western Blotting relative expression **Figure 1D**.

|  | Veh | Veh | Veh | Veh | Veh | TCS | TCS | TCS | TCS | TCS |
| --- | --- | --- | --- | --- | --- | --- | --- | --- | --- | --- |
| Fig. 1D UGT1A1 Liver | 1.00 | 0.27 | 0.02 | 0.12 | 0.05 | 24.47 | 8.26 | 11.41 | 8.03 | 8.07 |
| Fig. 1D GAPDH Liver | 1.00 | 1.04 | 1.01 | 1.13 | 0.81 | 1.07 | 1.09 | 0.88 | 0.76 | 0.75 |
| Fig. 1D UGT1A1 SI | 1.00 | 2.73 | 0.90 |  |  | 0.86 | 0.07 | 0.61 |  |  |
| Fig. 1D GAPDH SI | 1.00 | 0.98 | 0.83 |  |  | 0.85 | 0.91 | 0.96 |  |  |
|  |  |  |  |  |  |  |  |  |  |  |

**Supporting Table 12.** Western Blotting relative expression **Figure 2B**.

|  | Veh | Veh | Veh | TCS | TCS | TCS |
| --- | --- | --- | --- | --- | --- | --- |
| Fig. 2B CYP2B10 | 1.00 | 0.99 | 1.80 | 2.50 | 3.68 | 6.60 |
| Fig. 2B GAPDH | 1.00 | 0.83 | 0.90 | 0.87 | 0.61 | 0.63 |
| Fig. 2B NQO-1 | 1 | 1.271995 | 0.684176 | 6.923777 | 3.570057 | 5.432352 |
| Fig. 2B HO-1 | 1.00 | 0.58 | 1.05 | 1.88 | 1.41 | 2.44 |
| Fig. 2B GAPDH | 1.00 | 0.83 | 0.90 | 0.87 | 0.61 | 0.63 |

**Supporting Table 13.** Western Blotting relative expression **Figure 2C**. VC, Vehicle cytoplasm; TC, TCS cytoplasm; VN, Vehicle nucleus; TN, TCS nucleus.

|  | VC | VC | VC | VC | TC | TC | TC | TC | VN | VN | VN | VN | TN | TN | TN | TN |
| --- | --- | --- | --- | --- | --- | --- | --- | --- | --- | --- | --- | --- | --- | --- | --- | --- |
| Fig. 2C CAR | 1.00 | 1.30 | 1.19 | 1.46 | 1.43 | 1.32 | 2.51 | 2.82 | 0.66 | 0.64 | 1.17 | 1.28 | 4.56 | 4.05 | 3.99 | 1.14 |
| Fig. 2C HDAC2 | 1.00 | 1.48 | 1.68 | 1.01 | 0.65 | 0.72 | 0.72 | 0.97 | 4.29 | 4.88 | 6.51 | 6.33 | 6.63 | 7.34 | 3.05 | 8.66 |
| Fig. 2C TUBULIN | 1.00 | 0.93 | 0.91 | 0.92 | 0.86 | 0.92 | 0.83 | 0.87 | 0.01 | 0.01 | 0.03 | 0.05 | 0.20 | 0.21 | 0.19 | 0.10 |

**Supporting Table 14.** Western Blotting relative expression **Figure 3B and C**.

|  | Veh | Veh | TCS | TCS |
| --- | --- | --- | --- | --- |
| Fig. 3B UGT1A1 Liver | 1.00 | 0.86 | 4062.55 | 2167.01 |
| Fig. 3B GAPDH Liver | 1.00 | 0.73 | 0.86 | 1.22 |
| Fig. 3C UGT1A1 SI | 1.00 | 2.07 | 2.24 | 0.93 |
| Fig. 3C GAPDH SI | 1.00 | 2.07 | 2.38 | 1.83 |

**Supporting Table 15.** Western Blotting relative expression **Figure 4D and H**.

|  | *hUGT1* | | | | *hUGT1/Car^-/-^* | | | |
| --- | --- | --- | --- | --- | --- | --- | --- | --- |
|  | Veh | Veh | TCS | TCS | Veh | Veh | TCS | TCS |
| Fig. 4D UGT1A1 | 1.00 | 29.83 | 114.36 | 141.20 | 11.13 | 8.84 | 162.77 | 147.62 |
| Fig. 4D CYP2B10 | 1.00 | 0.84 | 1.52 | 1.15 | 0.01 | 0.01 | 0.01 | 0.02 |
| Fig. 4D GAPDH | 1.00 | 0.78 | 0.87 | 0.77 | 0.92 | 0.94 | 1.10 | 1.05 |
|  | *hUGT1* | | | | *hUGT1/Pparα^-/-^* | | | |
| Fig. 4H UGT1A1 | 1.00 | 0.90 | 157.05 | 101.83 | 6.50 | 9.50 | 5.25 | 6.18 |
| Fig. 4H GAPDH | 1.00 | 0.94 | 1.07 | 1.04 | 0.89 | 0.92 | 0.98 | 1.01 |

**Supporting Table 16.** Western Blotting relative expression **Figure 5A**.

|  | Veh | Veh | TCS | TCS |
| --- | --- | --- | --- | --- |
| Fig. 5A ATF4 | 1.00 | 1.26 | 7.93 | 4.71 |
| Fig. 5A CHOP | 1.00 | 1.36 | 2.16 | 4.52 |
| Fig. 5A GAPDH | 1.00 | 1.05 | 1.21 | 1.12 |

**Supporting Table 17.** Western Blotting relative expression **Figure 5D**.

|  | *hUGT1/Atf4^F/F^* | | | | *hUGT1/Atf4^ΔHep^* | | | |
| --- | --- | --- | --- | --- | --- | --- | --- | --- |
|  | Veh | Veh | TCS | TCS | Veh | Veh | TCS | TCS |
| Fig. 5D UGT1A1 | 1.00 | 1.22 | 179.30 | 90.41 | 3.79 | 4.04 | 1149.98 | 1483.90 |
| Fig. 5D GAPDH | 1.00 | 0.78 | 0.77 | 1.07 | 0.96 | 0.93 | 1.05 | 0.84 |
